# Supplementary material for: Adaptive microwave impedance memory effect in a ferromagnetic insulator
Source: Nat Commun. 2016 Dec 14;7:13737. doi: 10.1038/ncomms13737 (PMC5477504; doi:10.1038/ncomms13737)
Supplement: Supplementary Information — Supplementary Figures 1-17, Supplementary Notes 1-7 and Supplementary References. [file ncomms13737-s1.pdf]

SUPPLEMENTARY FIGURES

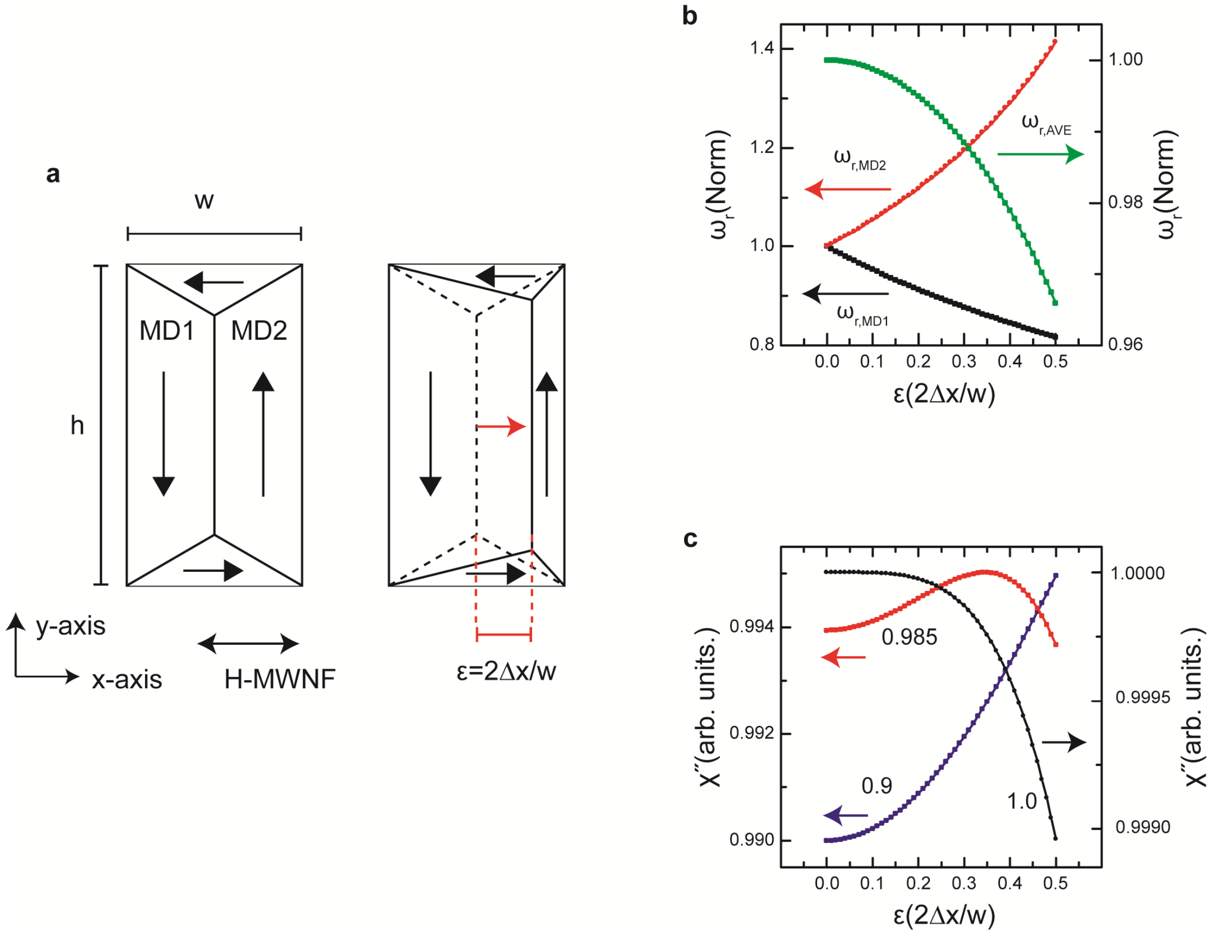

Supplementary Figure 1**: Illustration of the relation between the magnetic domain structure and the magnetic susceptibility.** (**a**) Illustration of the magnetic domains forming the Landau flux-closure structure. The black arrows indicate the magnetization direction of the magnetic domains, and red arrow indicates a shift of domain wall (ε). (**b**) Calculated natural ferromagnetic resonance (N-FMR) frequency changes as a function of domain wall shift, where $\omega_{\mathrm{r,MD1}}$ (black) and $\omega_{\mathrm{r,MD2}}$ (red) are the N-FMR frequency of the MD1 and the MD2, and $\omega_{\mathrm{r,AVE}}$ (green) is the effective N-FMR frequency of the MD structure. (**c**) Calculated imaginary magnetic susceptibility (χ'') as a function of domain wall shift for probing microwave frequencies: $\omega/\omega_{\mathrm{r,}}$=1.0 (black), 0.985 (red), and 0.9 (blue).

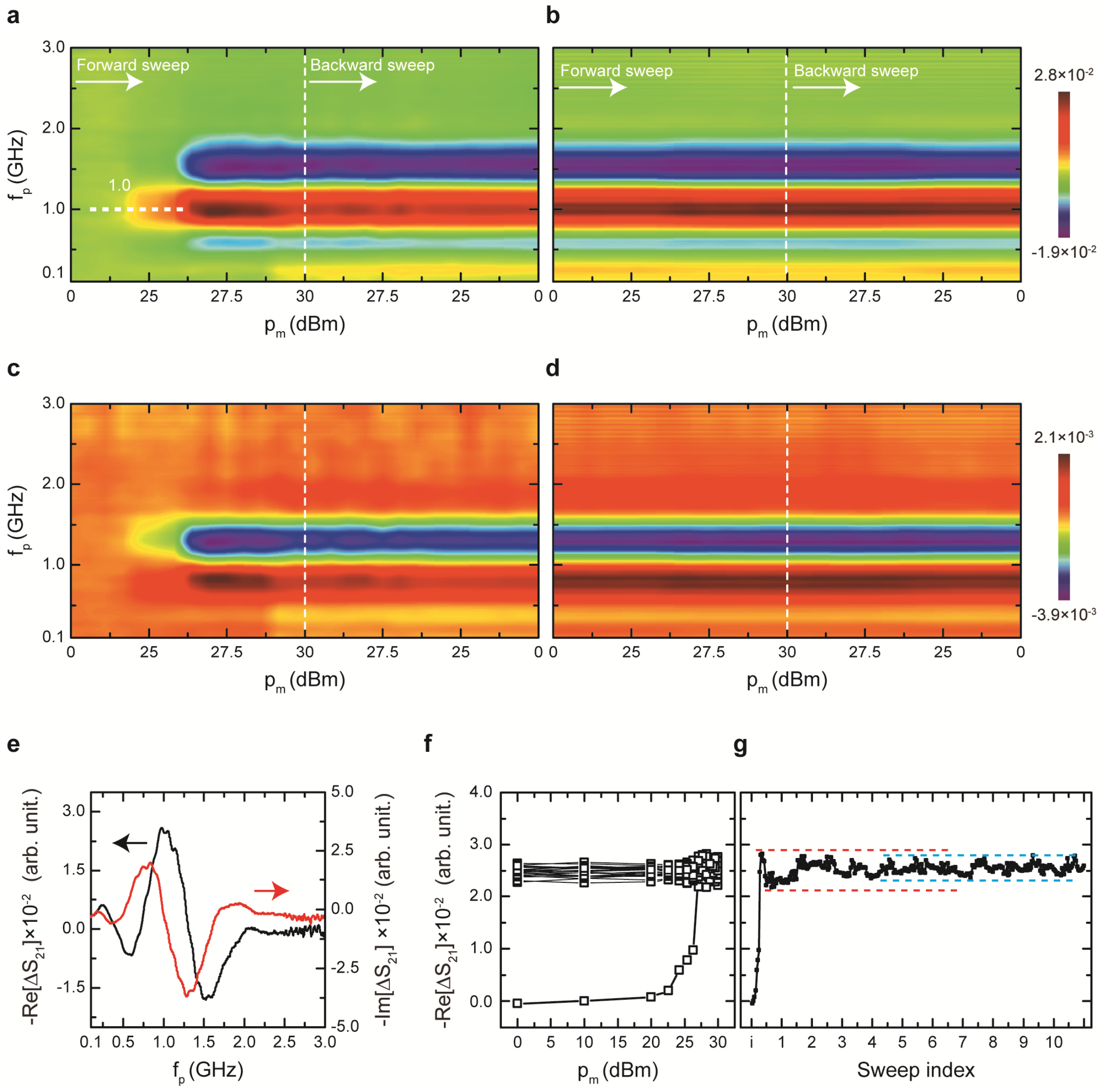

Supplementary Figure 2**: Microwave response modulation by p_m-sweep.** (**a-d**) Real (**a**, **b**) and imaginary (**c**, **d**) parts of $\Delta S_{21,p}$ as a function of p_m for f_m=1.0 GHz measured at the initial sweep (**a**, **c**) and averaged (10-time) after the initial sweep (**b**, **d**). (**e**) Line-profiles of real (black) and imaginary (red) parts of the $\Delta S_{21,p}$ at p_m=30 dBm. (**f-g**) Real part of the $\Delta S_{21,p}$ for a fixed f_p=1.0 GHz as a function of p_m (**f**) and sweep index (**g**). The dashed lines in **g** indicate a decrease of fluctuation of the $\Delta S_{21,p}$ change.

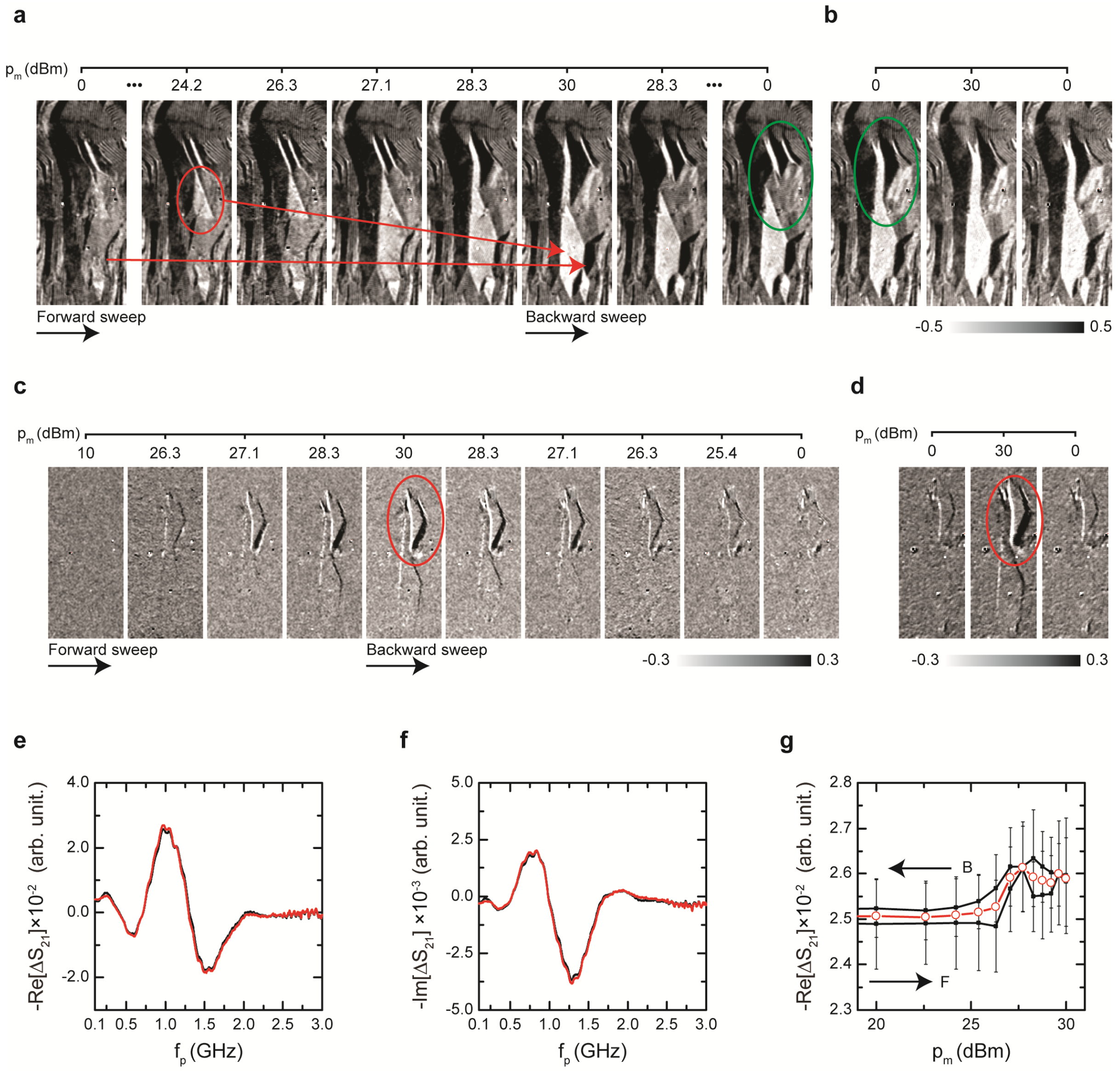

Supplementary Figure 3**: Changes of the magnetic domain structure by p_m-sweep. (a-b)** MD images measured at the initial p_m-sweep (**a**) and at the tenth p_m-sweep (**b**). The red arrows indicate initial MDs that remained after the p_m-sweep, and green circles indicate a region showing a change of MDs by subsequent p_m-sweep. (**c**) Difference of MD images at tenth p_m-sweep from initial MD images measured at p_m= 0dBm. (**d**) Averaged difference of MD images after the initial p_m-sweep. The red circle indicates a region showing a reversible change of MDs during the p_m-sweep. (**e-f**) Line-profiles of real (**e**) and imaginary (**f**) $\Delta S_{21,p}$ measured at p_m=0 dBm (black) and 30 dBm (red) in tenth p_m-sweep. **g.** Averaged real $\Delta S_{21,p}$ changes at f_p=1.0 GHz as a function of p_m. The red line is the averaged $\Delta S_{21,p}$ of forward (F) and backward (B) p_m-sweeps. The error bars represent the standard deviation of the averaged data.

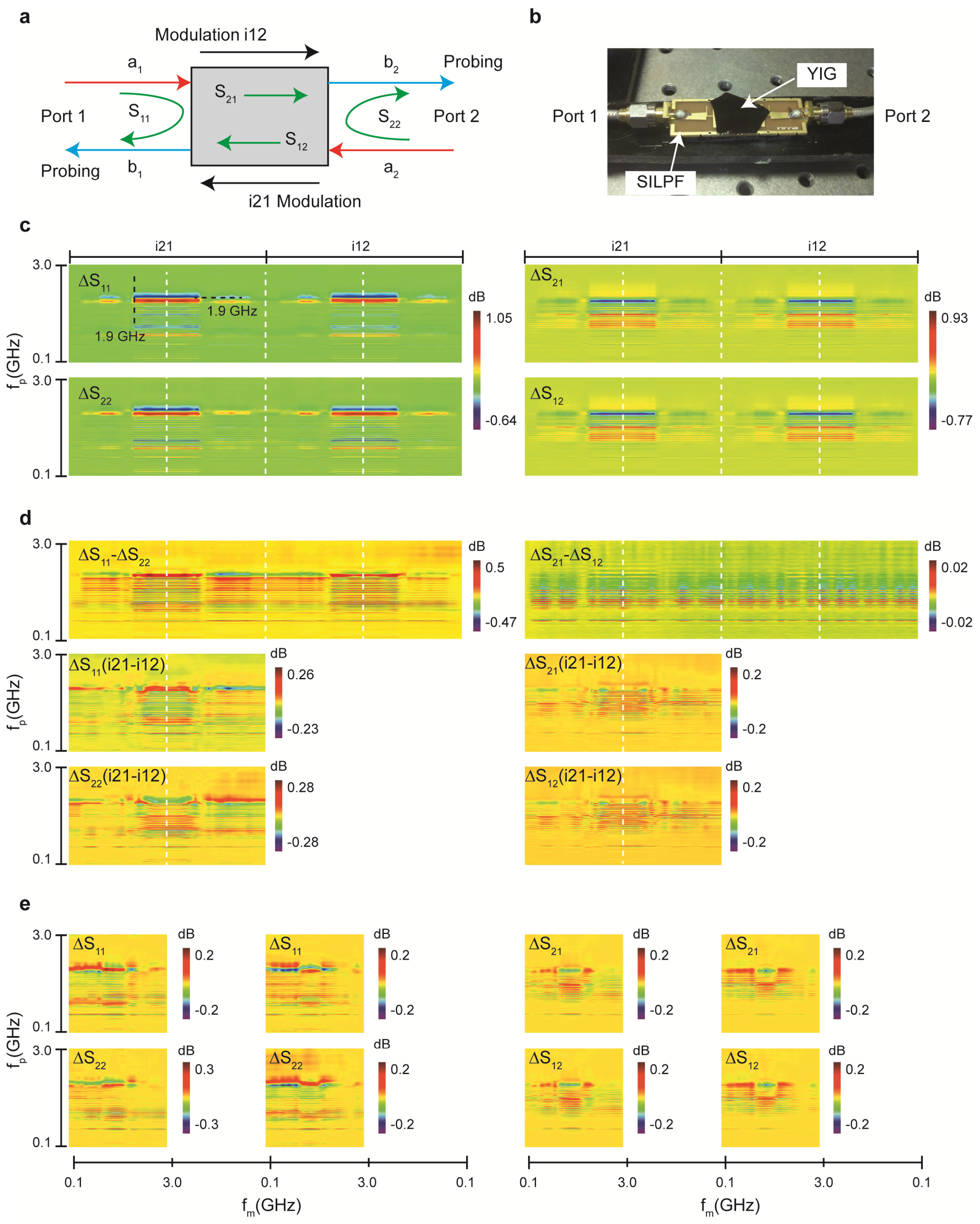

Supplementary Figure 4**: Changes of *S*-parameters by f_m-sweep.** (**a**) Illustration of *S*-parameters for the two ports device, where a_1 and a_2 denote incident probing microwave for port 1 and port 2, and b_1 and b_2 denote reflected and transmitted probing microwaves, and i12 and i21 denote the applied direction of the modulation microwaves. (**b**) Optical image of the experimental setup, where the device was connected to a network analyzer (E5071B; Agilent)

by 50Ω BNC cables. (**c**) Contour maps of averaged changes of *S*-parameters as a function of probing (f_p) and modulation (f_m) microwave frequency, where the power of probing (p_p) and modulation (p_m) microwaves were -20 dBm and 10 dBm, respectively, and the excitation time was 0.1 s. The *S*-parameters were averaged for 9-modulation loops after initial modulation loop. (**d**) Reciprocity of the device for probing and excitation process, where the reciprocity means that the device response is not changed when the ports of the device are swapped with each other. (**e**) Hysteresis of S-parameters between forward and backward modulation sweeps.

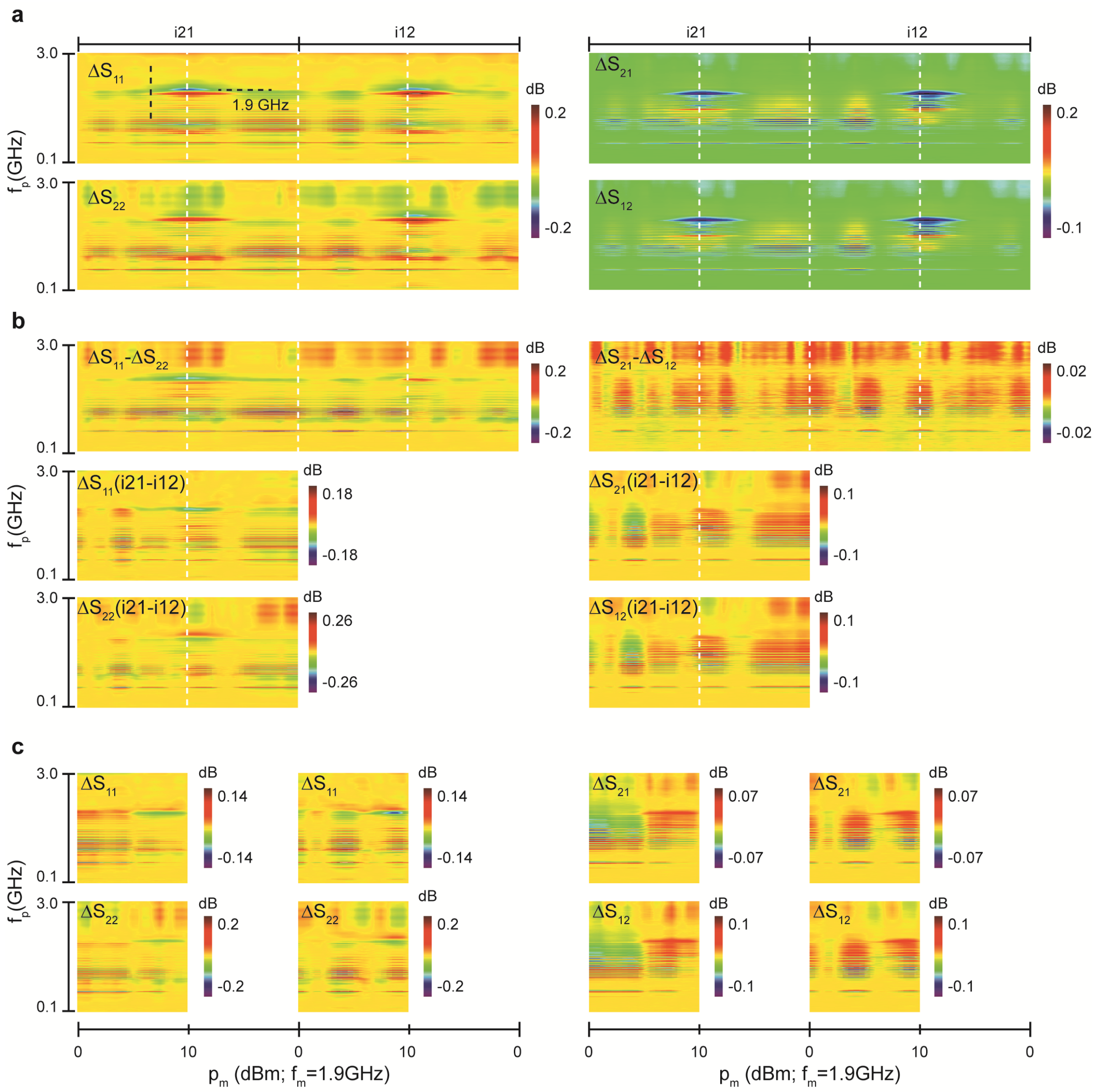

Supplementary Figure 5**: Changes of *S*-parameters by p_{m}-sweep.** (**a**) Contour maps of averaged changes of *S*-parameters as a function of probing microwave frequency (f_{p}) and modulation microwave power (p_{m}), where the power of probing microwave (p_{p}) was -20 dBm, and modulation microwave frequency was 1.9 GHz, and the excitation time was 0.1 s. The *S*-parameters were averaged for 9-modulation loops after initial modulation loop. **b.** Reciprocity of the device. **c.** Hysteresis of *S*-parameters.

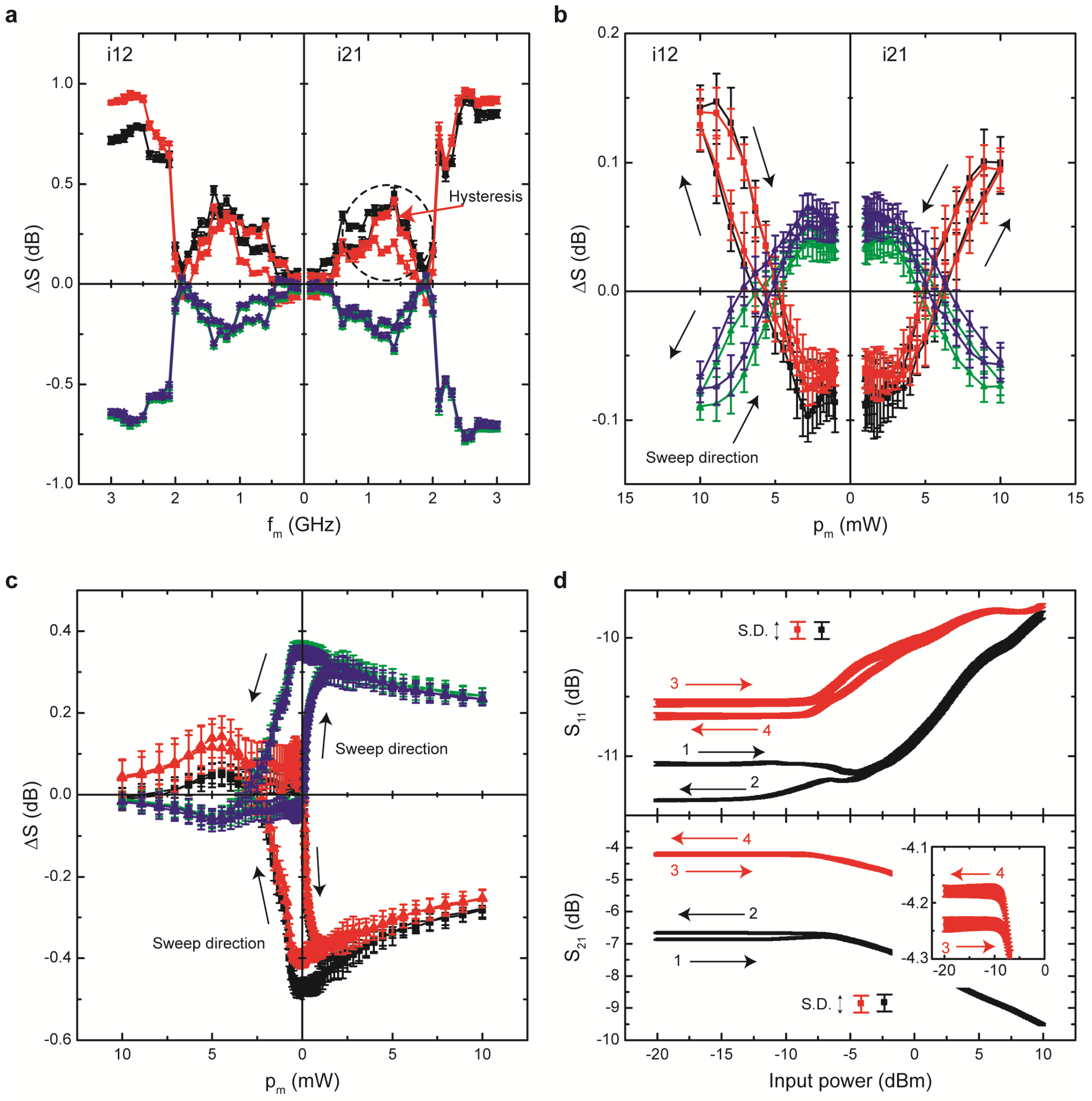

Supplementary Figure 6**: Detailed structures of *S*-parameters changes by the modulation microwave.** (**a**) Changes of *S*-parameters (black:ΔS_{11}; red: ΔS_{22}; green: ΔS_{12}; blue: ΔS_{22}) as a function of modulation microwave frequency, where the probing frequency (f_p) and power (p_p) were 1.9 GHz and -20 dBm, and modulation microwave power (p_m) was 10 dBm. (**b**) Changes of *S*-parameters as a function of modulation microwave power, where the error bar indicates the standard deviation of the averaged measurement results. The probing frequency (f_p) and power (p_p) were 1.9 GHz and -20 dBm, and the modulation microwave frequency (f_m) was 1.9 GHz. The arrows indicate directions of the modulation sweeps. (**c**) Implementation of continuously tunable bi-stable state memory operation by two different modulation microwave frequencies (1.9 GHz and 2.1 GHz), where the probing microwave frequency (f_p)

and power (p_p) were 1.9 GHz and -20 dBm. The changes of *S*-parameters for 2.1 GHz and 1.9 GHz excitation frequency appear in the left and right side of the chart, respectively. (**d**) Changes of S_{11} and S_{21} as a function of applied microwave power with a frequency of f=1.9 GHz (black) and f=2.1 GHz (red), where the error bar indicates the standard deviation of the measurement results.. The arrows and numbers denote the sweep direction and sequence: from 1 (increasing power; forward) to 2 (decreasing power; backward) at 1.9 GHz; from 3 (forward) to 4 (backward) at 2.1 GHz. The measurement loops (1-4-1) were conducted for 10 times, where the power was increased from -20 dBm to 10 dBm with a step of 0.1 dBm. For each measurement steps, the microwave was applied for a short time less than 0.1 seconds, and a cooling time (~3 seconds) was introduced between the steps.

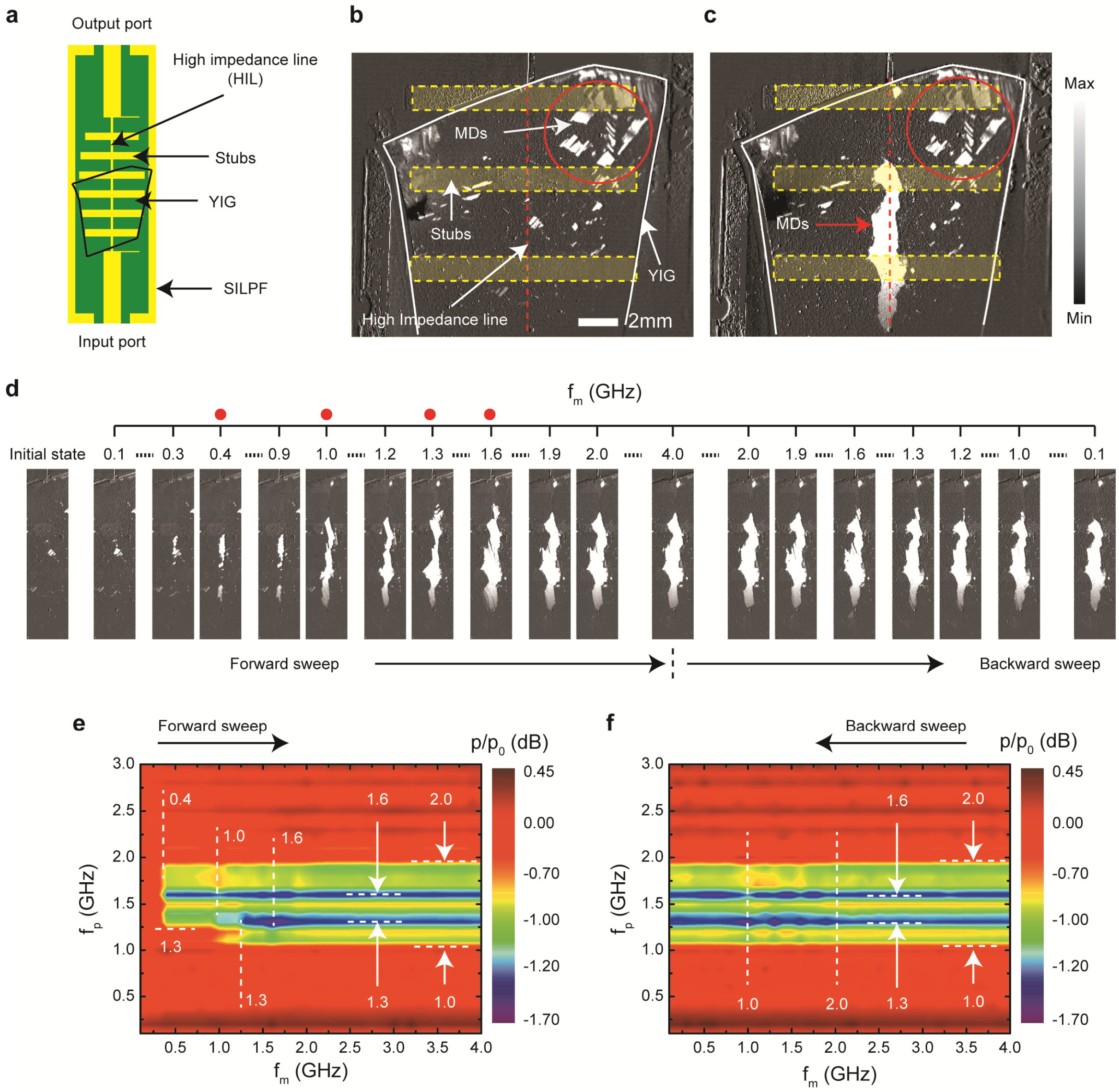

Supplementary Figure 7**: Measurement results for initial f_m-sweep in SILPF/YIG/GGG-configuration.** (**a**) Illustration of the measurement setup. (**b-c**) Magnetic domain images measured before (**b**) and after (**c**) microwave excitations. (**d**) Changes of magnetic domain structure as a function of modulation microwave frequency (f_m). (**e-f**) Contour maps of microwave transmittance as a function of modulation (f_m) and probing (f_p) frequencies for forward (**e**) and backward (**f**) f_m-sweep, where p_0 is the transmitted power measured before the modulation sweep.

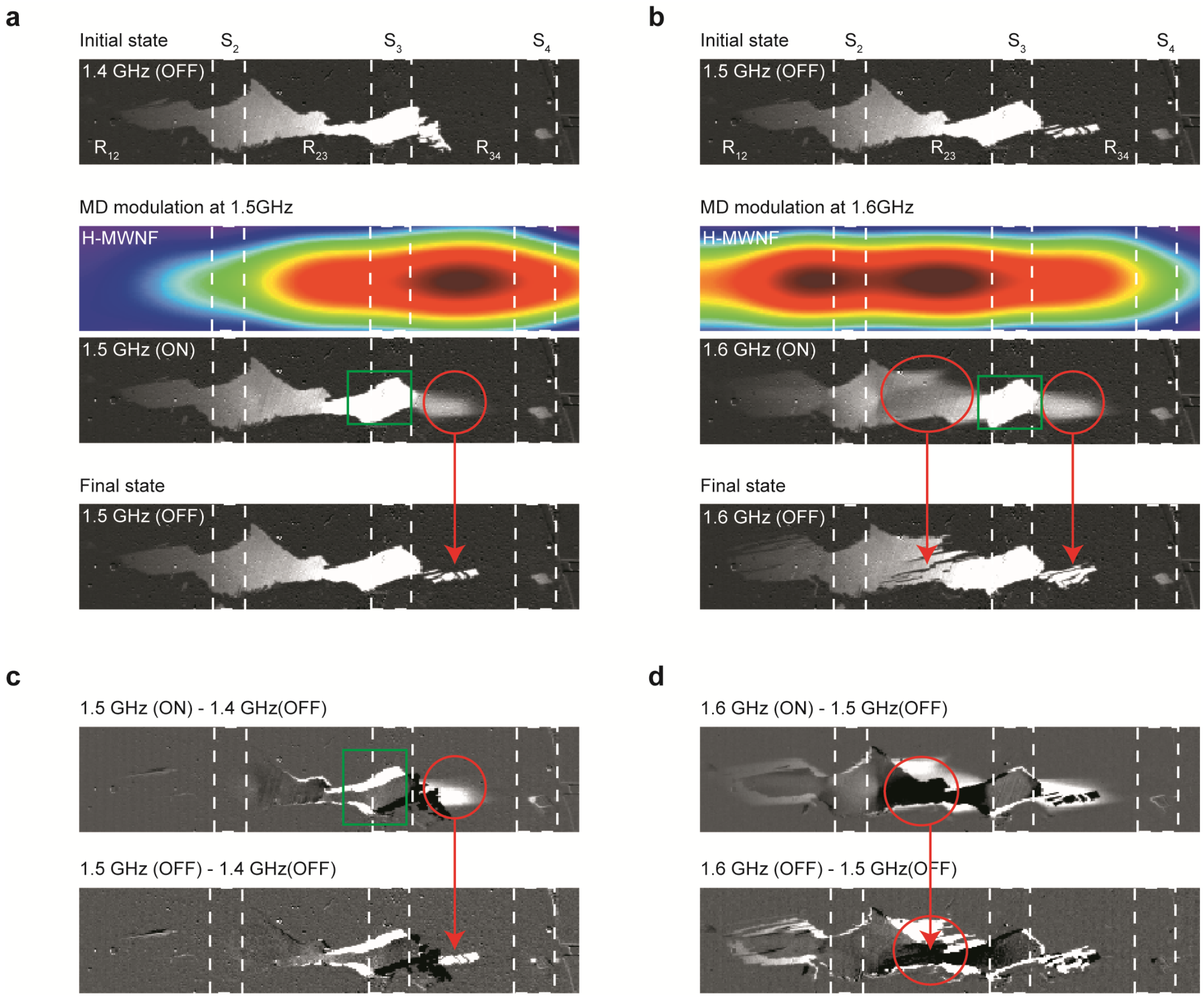

Supplementary Figure 8**: Detailed process of MD modulation occurring in excitations from 1.4 GHz to 1.6 GHz.** Magnetic domain (gray) and H-MWNF (color) images during the initial excitation loop: (**a**) from 1.4 GHz to 1.5 GHz; (**b**) from 1.5 GHz to 1.6 GHz. The ON and OFF denote that the images were measured under applying excitation MW (ON) or measured after releasing the excitation MW (OFF). (**c-d**) Differences of magnetic domain structure between the initial state and during the excitations, and between the initial states and after the excitations. The green rectangles indicate regions showing a distinct change of MD structure, and red circles indicate regions showing an appearance of a new MD structure that fluctuated after the excitations.

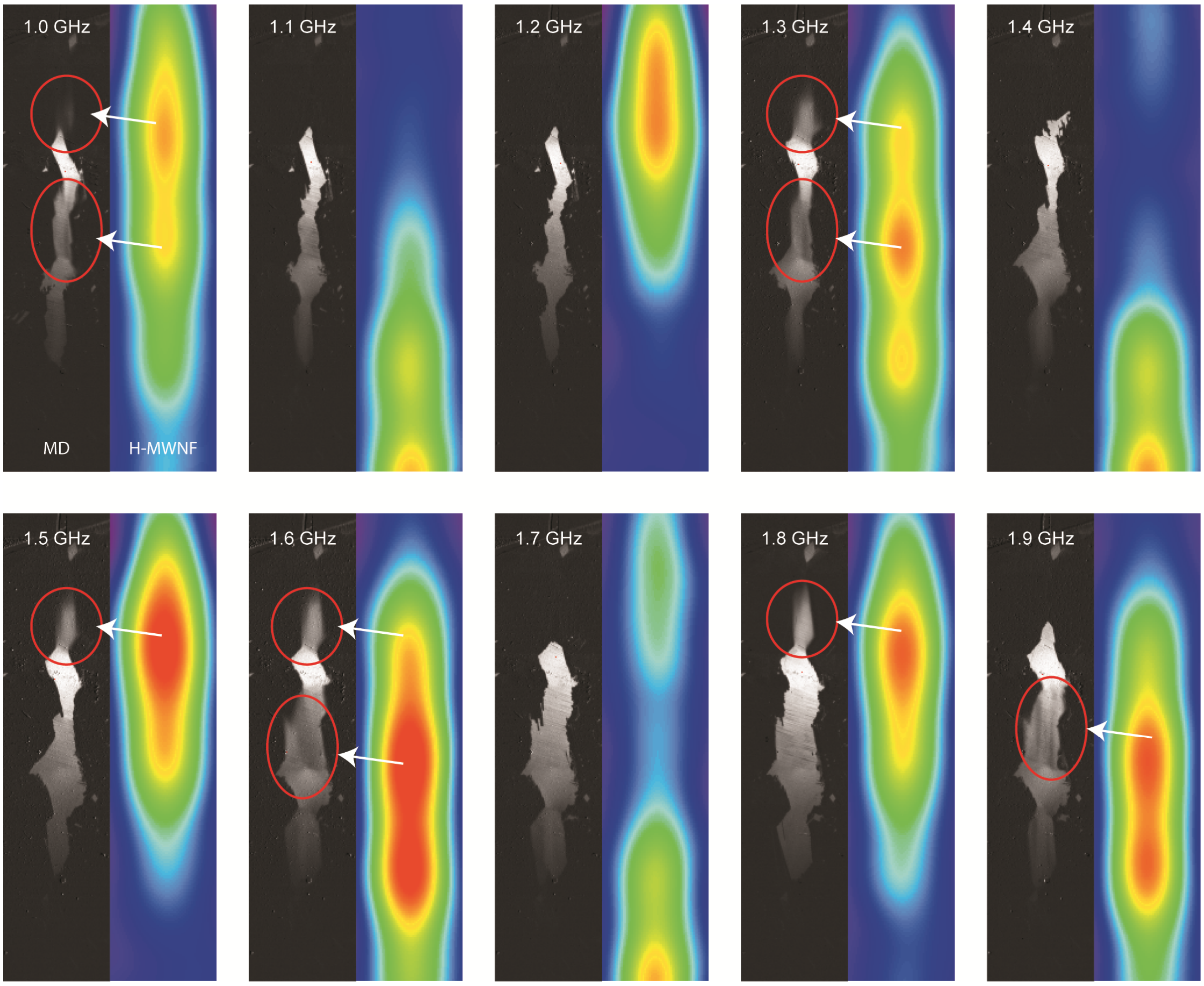

Supplementary Figure 9**: Magnetic domain structures under modulation microwave signals.** Magnetic domain (gray images) and magnetic microwave near field (color images) images measured as a function of modulation frequency. Indistinct magneto optical (MO) signals (indicated by red circles) appeared around intense magnetic microwave near field regions (indicated by white arrows). The coincident of the regions showing a blurred MO signal and an intense magnetic microwave near field indicates a structural instability of the magnetic domains caused by a strong spin precession.

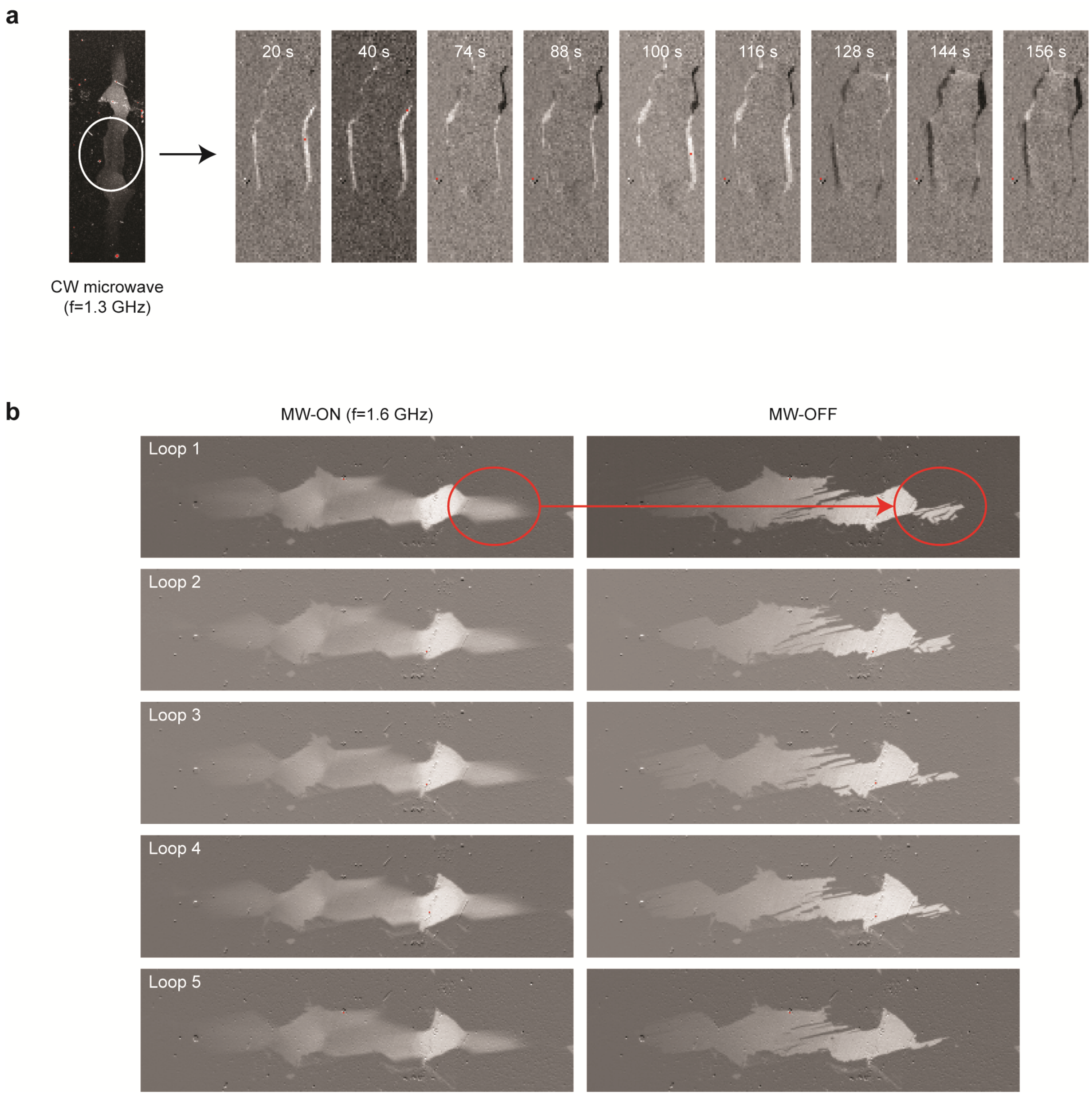

Supplementary Figure 10**: Fluctuations of a magnetic domain structure by modulation microwave signals.** (**a**) Changes of magnetic domain structure as a function of elapsed time, where the continuous microwave of 1.3 GHz was applied during the measurement. The images were calculated by subtracting the image measured at t=0s. The changes of magnetic domain structure indicate that there is a structural instability of the modulated magnetic domain structure. (**b**) Magnetic domain structures for different excitation loops measured at turn-on and turn-off microwave excitation states at 1.6 GHz. One can see that a new magnetic domain structure appears for each excitation loops on the region showing a blurred MO signal (indicated by red circle). This indicates that the magnetic domains strongly fluctuate at the region, and there is a structural instability of the magnetic domain structure.

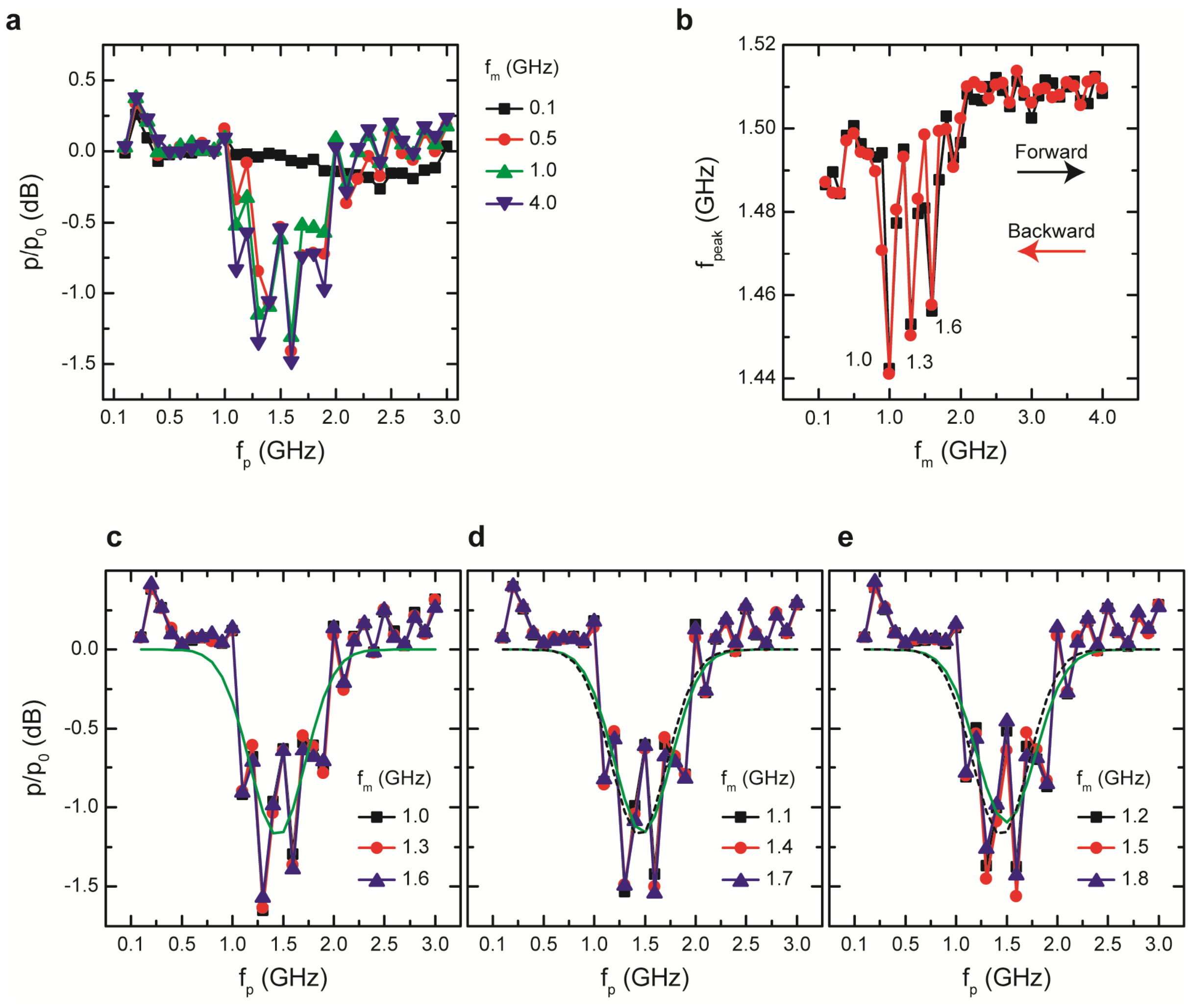

Supplementary Figure 11**: Changes of the N-FMR frequency by the f_m-sweep in SILPF/YIG/GGG-configuration.** (**a**) Line profiles of microwave transmittance for f_m=0.1, 0.5, 1.0 and 4.0 GHz during the forward initial f_m-sweep. (**b**) Calculated N-FMR frequencies (f_{peak}) after the initial f_m-sweep. (**c-e**) Line profiles of microwave transmittance for various f_m: (**c**) f_m=1.0, 1.3 and 1.6 GHz; (**d**) 1.1, 1.4 and 1.7 GHz; (**e**) 1.2, 1.5, 1.8 GHz. The fitting curves (green) for the N-FMR frequency calculation are presented with the line-profiles, where the dashed black curves in d-e shows the fitting curve of figure (**c**).

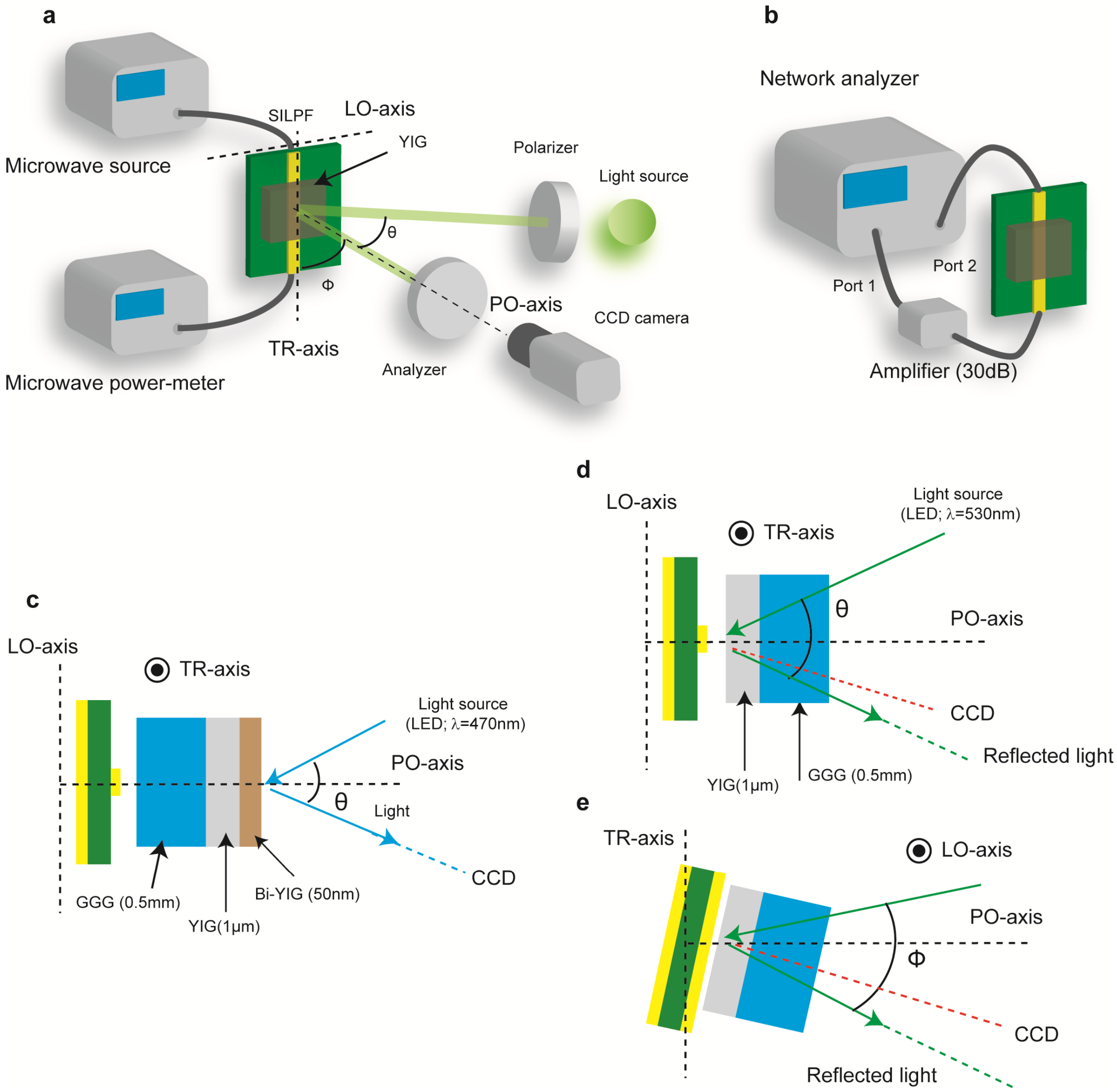

Supplementary Figure 12: **Illustrations of the microwave measurement setup and the magneto optical imaging.** (**a**) SILPF/YIG/GGG-configuration for the microwave transmittance measurement. (**b**) SILPF/GGG/YIG/BI-YIG-configuration for the S-parameter measurement. (**c**) Illustration of transverse magneto optical (MO) Kerr effect measurement for the SILPF/GGG/YIG/BI-YIG-configuration. The Bi-YIG layer was monitored by the MO imaging system, where red arrow indicates the polarization direction of the incident light, and cyan arrows indicate the propagation direction of the incident light (λ=470nm). (**d-e**) Illustrations of the MO imaging setup for SILPF/YIG/GGG-configuration along the TR (**d**) and LO (**e**) axis, where the CCD was slightly deviated from the propagation direction of the reflected light, and a green wavelength (λ=530nm) was used for the light source.

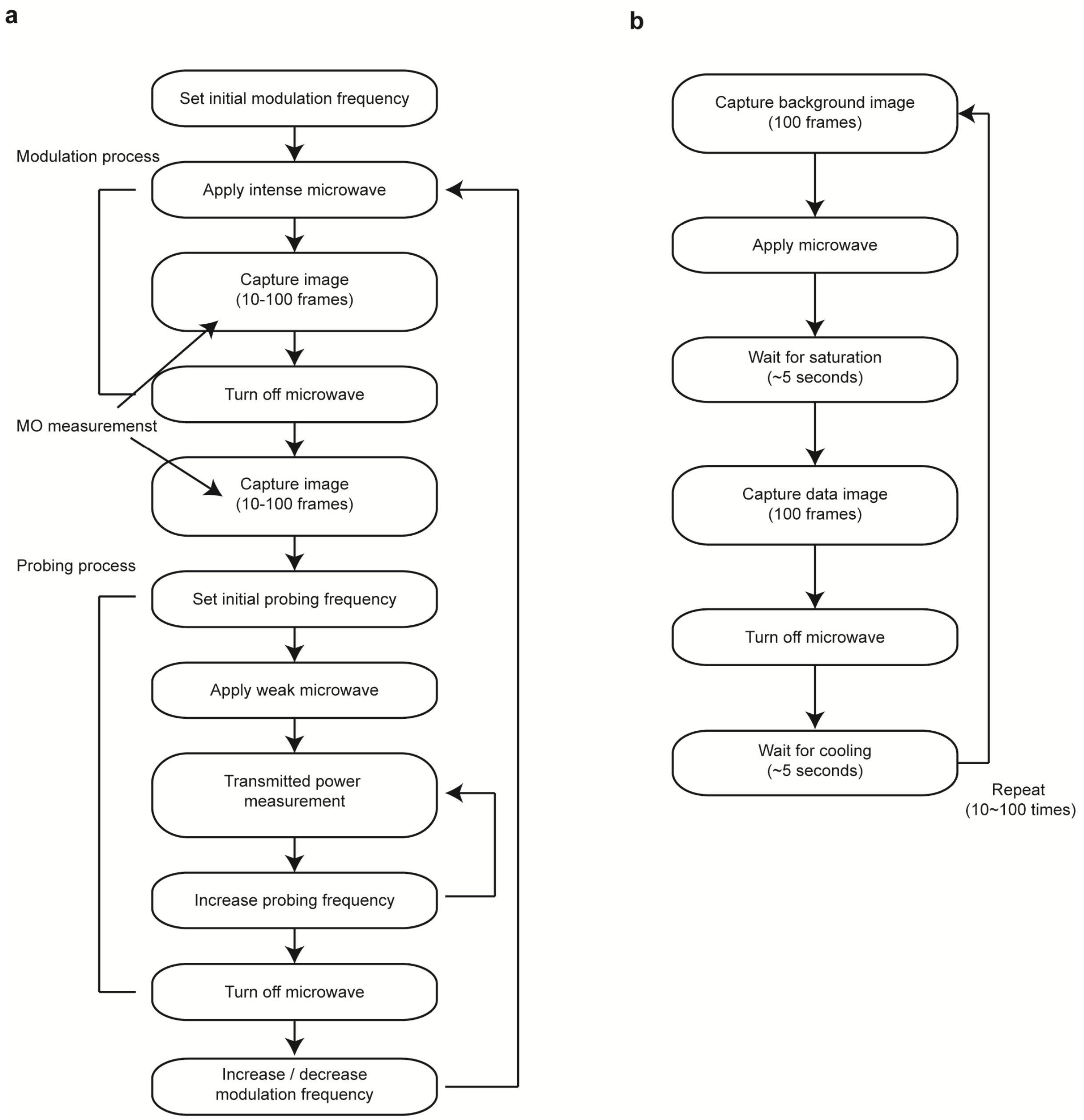

Supplementary Figure 13**: Flowcharts of measurement procedures.** (**a**) Measurement process of MO imaging and microwave transmittance measurements. (**b**) Measurement process of magnetic microwave near field (H-MWNF) imaging.

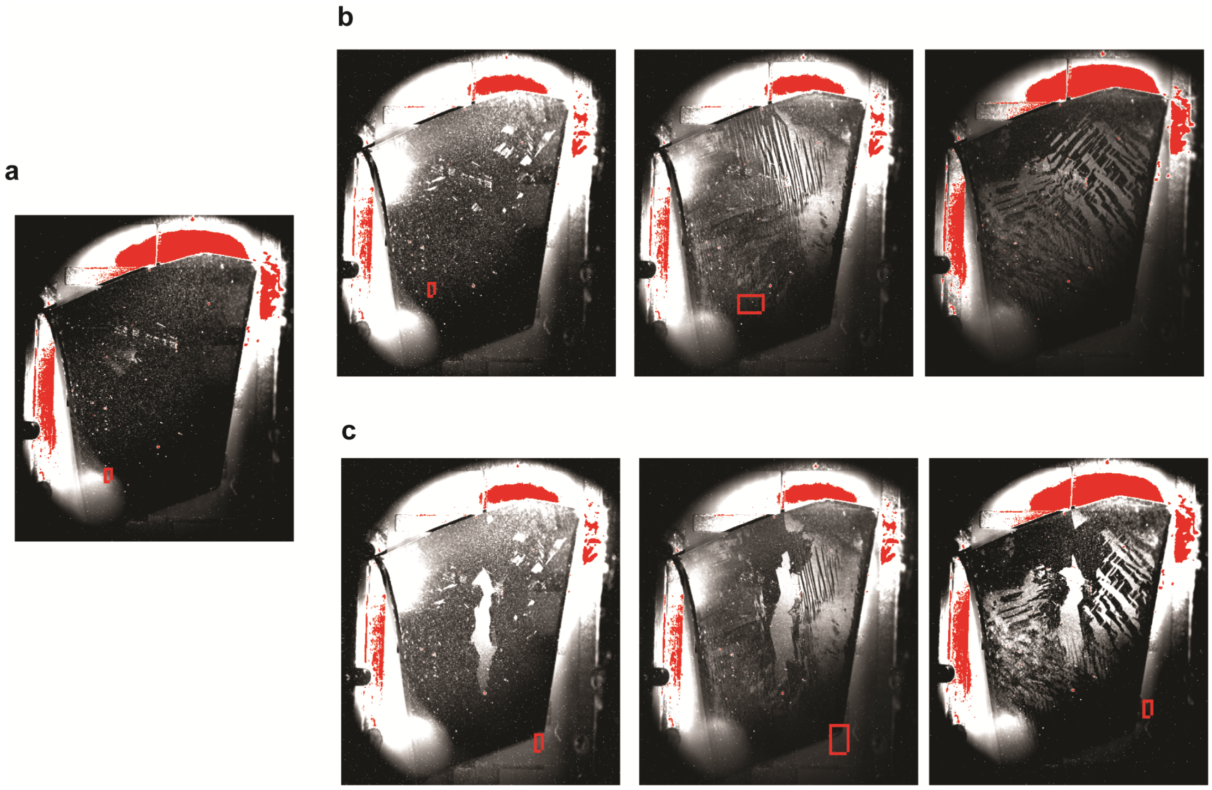

Supplementary Figure 14**: Representative magneto-optical measurement results in SILPF/YIG/GGG-configuration.** The images were measured with (**a**) and without external magnetic field (**b**), and after a microwave excitation (**c**).

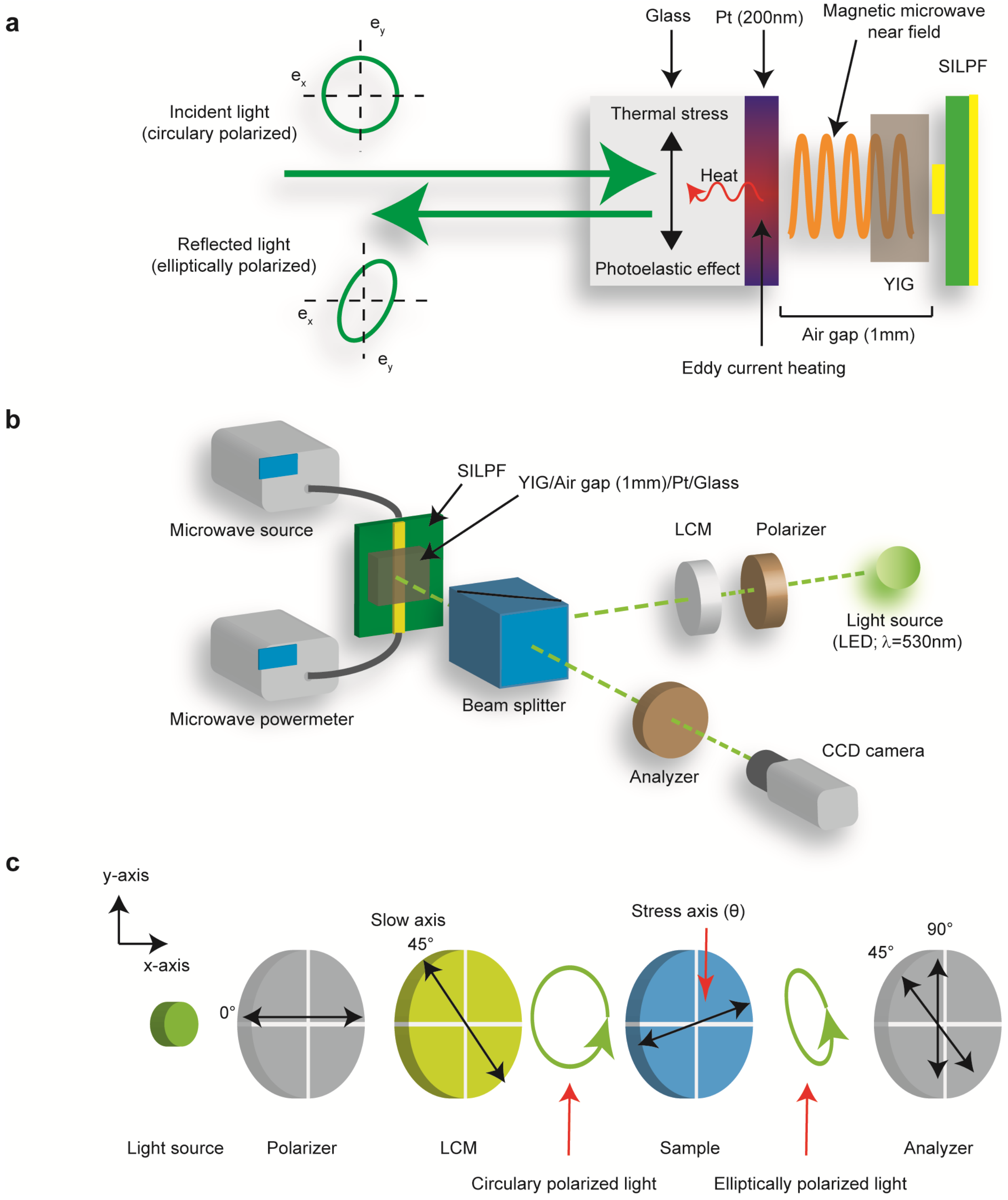

Supplementary Figure 15: **Illustrations of the magnetic microwave near field measurement.** (**a**) Illustrations of measurement principle of the magnetic microwave near field (H-MWNF) by the photoelastic optical indicator method. (**b**) Illustration of experimental setup for the H-MWNF imaging. (**c**) Optical axis configurations of optical components.

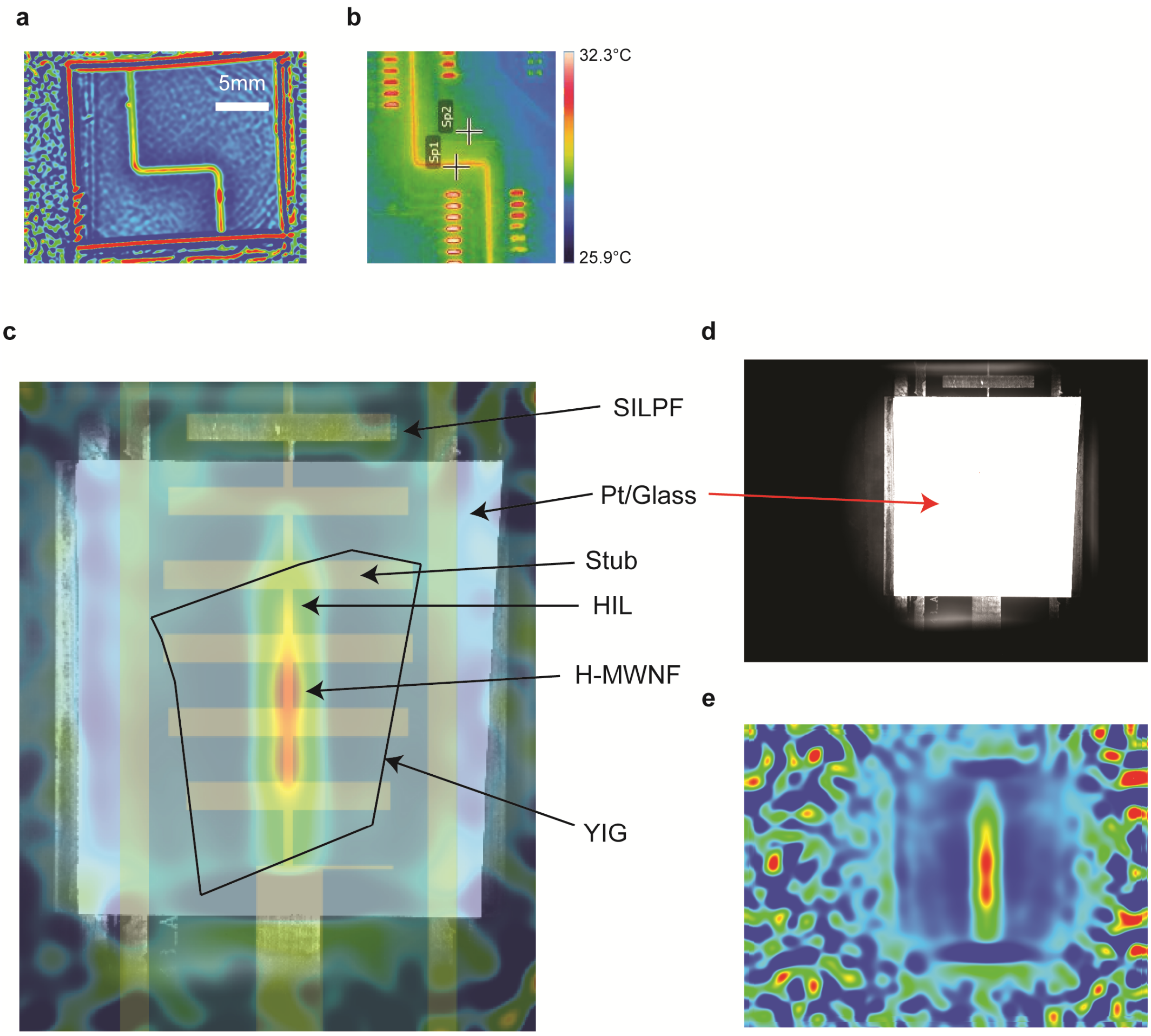

Supplementary Figure 16**: Temperature and magnetic microwave near field images measured by the photoelastic optical indicator method.** (**a-b**) Calculated heat distribution image by the present technique (**a**) and temperature distribution image measured by an IR-camera (**b**), where a PCB circuit was used as the device under test. (**c**) Magnetic microwave near field (H-MWNF) distribution image of stepped impedance low pass filter (SILPF) measured by the present technique. (**d**) Optical image of the device under test. (**d**) Calculated H-MWNF distribution image.

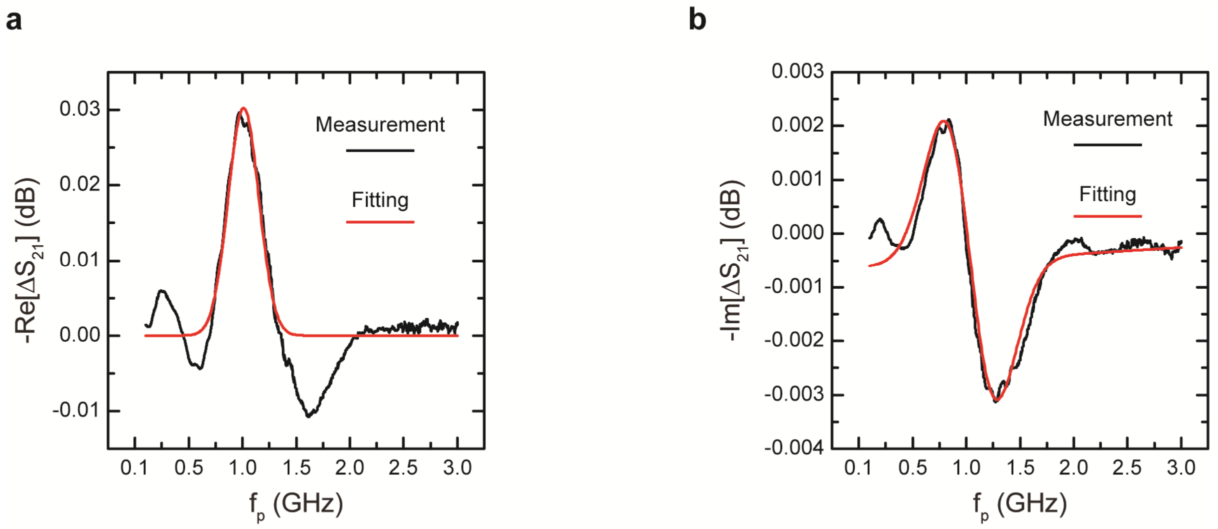

Supplementary Figure 17**: Representative results of curve fitting for the N-FMR frequency calculation.** (**a**) Real part of the ΔS_{21}. (**b**) Imaginary part of the ΔS_{21}.

SUPPLEMENTARY NOTES

Supplementary Note 1: L-model for 2-port N-FMR device.

In present study, the microwave response is governed by a ferromagnetic material coupled with a high impedance line (L»C) of the stepped impedance low pass filter (SILPF), and thus, one can simplify as that the impedance change mainly comes from a change of inductance of the device. The inductance change is a function of magnetic susceptibility of the coupled magnetic material as below[1]:

$$Z \cong i\omega L = C(\chi'' + i\chi'), \text{ where } \chi = \chi' - i\chi'', \text{ (Supplementary Equation 1)}$$

where, χ' and χ'' are the real and imaginary parts of dynamic magnetic susceptibility, and C is a constant related to a structure of waveguide and a coupling strength. Here, we focus on a system that can be described as a two port device connected in series with the waveguide system, where the device has variable impedance Z_s. For an incident microwave at port 1, two S-parameters S_{11} and S_{21} describe the reflected and transmitted microwave signals, and they can be found from the ABCD-parameters as below[2]:

$$ABCD = \begin{bmatrix} A & B \\ C & D \end{bmatrix} = \begin{bmatrix} 1 & Z_s \\ 0 & 1 \end{bmatrix}, \text{ (Supplementary Equation 2)}$$

$$S_{11}=\frac{A+B/Z_0-CZ_0-D}{A+B/Z_0-CZ_0+D}=\frac{Z_s/Z_0}{2+Z_s/Z_0},\quad S_{21}=\frac{2}{A+B/Z_0+CZ_0+D}=\frac{1}{1+Z_s/2Z_0},$$

(Supplementary Equation 3)

where, the Z_0 is the characteristic impedance across all ports. By taking a weak reflection approximation (S_{11}«1 and $Z_s/2Z_0$«1), one can find simplified expressions as below:

$$\log S_{21}=-\log\left[1+\frac{Z_s}{2Z_0}\right]\cong-\frac{Z_s}{2Z_0},$$ (Supplementary Equation 4)

$$S_{21}=\exp\left[-\frac{Z_s}{2Z_0}\right],\ \text{and}\ \ S_{11}=1-S_{21},$$ (Supplementary Equation 5)

Then, the incident and transmitted/reflected microwaves of the system are related as below:

$$b_t^{(s)}=S_{21}^{(s)}a=\exp\left[-\frac{Z_s}{2Z_0}\right]a,$$ (Supplementary Equation 6)

$$b_r^{(s)}=S_{11}^{(s)}a=\left[1-\exp\left[-\frac{Z_s}{2Z_0}\right]\right]a,$$ (Supplementary Equation 7)

From supplementary equations (6)-(7), one can find the modulated amplitude and phase of the transmitted microwave by the variable impedance as below:

$$\Delta A\sim\log\frac{b_0^{(s)}}{a_0}=-\mathrm{Re}\left\{\log S_{21}^{(s)}\right\}=\mathrm{Re}\left\{\frac{Z_s}{2Z_0}\right\}\sim\chi'',$$ (Supplementary Equation 8)

$$\Delta\varphi\sim\left(\varphi_b-\varphi_a\right)=-\mathrm{Im}\left\{\log S_{21}^{(s)}\right\}=\mathrm{Im}\left\{\frac{Z_s}{2Z_0}\right\}\sim\chi',$$ (Supplementary Equation 9)

where, ΔA and $\Delta\varphi$ are the changes of amplitude and phase of the transmitted microwave, and the incident and transmitted microwaves are described as below:

$$a(a_0,\omega,\varphi_a)=a_0e^{i(\omega t+\varphi_a)},b(b_0,\omega,\varphi_b)=b_0e^{i(\omega t+\varphi_b)},$$ (Supplementary Equation 10)

The supplementary equations (8)-(9) imply that the change of real and imaginary part of the

impedance of the system will modulate the amplitude and phase of an incident microwave, respectively, and one can find these modulations using the experimentally accessible S-parameters.

The dynamic magnetic susceptibility is related to the dynamical behavior of magnetization (**M**) in the magnetic material, and the behavior can be described by the Landau-Lifshitz-Gilbert (LLG) equation[3]:

$$\frac{\partial \mathbf{M}}{\partial t} = -\gamma \mathbf{M} \times \mathbf{H}_{eff} + \frac{\alpha}{M_{\mathrm{s}}} \mathbf{M} \times \frac{\partial \mathbf{M}}{\partial t}, \quad \mathbf{H}_{\mathrm{eff}} = \mathbf{H}_{\mathrm{i}} + \mathbf{h}_{\mathrm{rf}} \text{ (Supplementary Equation 11)}$$

where, M_{s} is the saturation magnetization, γ is the gyromagnetic ratio, and α is the damping constant, $\mathbf{h}_{\mathrm{rf}}$ is the oscillating magnetic field, and $\mathbf{H}_{\mathrm{i}}$ is the total effective field that includes anisotropic field, demagnetization field, static external magnetic field, and so on. For a ferromagnetic material magnetized by a static external magnetic field, a weak oscillating magnetic field perpendicular to the static magnetic field exerts a torque on the magnetization, and the magnetic moment start to precess around the static magnetic field. The ferromagnetic resonance (FMR) occurs when the driving frequency is close to the natural frequency of the precessional motion (Lamor frequency), and the resonance frequency depends on the external magnetic field, magnetization state and magnetic property of the material. As a result, the induced magnetic moment by an oscillating magnetic field will show a resonant behavior, and one can express the dynamic susceptibility around the resonance frequency as:

$$\chi\left(\omega \approx \omega_{\mathrm{r}}\right) \equiv \frac{M_{\mathrm{rf}}}{h_{\mathrm{rf}}} \sim -\frac{\chi_0}{2} \frac{\omega_{\mathrm{r}}}{\omega - \omega_{\mathrm{r}} + i\Gamma/2}, \Gamma = 2\alpha\eta, \text{ (Supplementary Equation 12)}$$

where, ω_{r} is the resonance frequency, χ_0 is the static magnetic susceptibility, Γ is the line width of the resonance which is a function of the damping constant α and the damping factor η. Then, one can find the real and imaginary parts of the dynamic susceptibility as:

$$\chi'(\omega) = \frac{\chi_0}{2} \frac{\omega_r\left(\omega - \omega_r\right)}{\left(\omega - \omega_r\right)^2 + \left(\Gamma/2\right)^2}, \chi''(\omega) = \frac{\chi_0}{2} \frac{\omega_r\left(\Gamma/2\right)}{\left(\omega - \omega_r\right)^2 + \left(\Gamma/2\right)^2}, \text{ (Supplementary Equation 13)}$$

The imaginary part of the susceptibility is maximized around the resonance frequency with a Lorentzian line shape, while the real part of the susceptibility shows an anomalous dispersion around the resonance frequency. The supplementary equation (13) can be simplified by taking

the damping factor η to be a linear function of resonance frequency ($\eta \sim k\omega_0$):

$$\chi' \approx \frac{\chi_0}{2\alpha^2 k^2}(1-s)\,,\chi'' \approx \frac{\chi_0}{2\alpha^2 k^2}\left[1-\left(\frac{1-s}{\alpha k}\right)^2\right],\ \ s=\frac{\omega}{\omega_r}\text{, (Supplementary Equation 14)}$$

One can calculate a change of susceptibility for a fixed probing frequency about a variation of the resonance frequency ($\omega_r'=\omega_r+\Delta\,\omega_r$) as:

$$\Delta\chi' = \frac{\chi_0}{2\alpha^2 k^2}\frac{\Delta\omega_r}{\omega_r}s\,,\Delta\chi'' = \frac{\chi_0}{2\alpha^2 k^2}\frac{\Delta\omega_r}{\omega_r}s(1-s)\text{, where }\Delta\chi = \frac{d\chi}{d\omega_r}\Delta\omega_r\,,$$

(Supplementary Equation 15)

The resonance frequency can be found from the magnetic free energy (F) of the material by the following equation[3]:

$$\omega_r = \frac{\gamma}{M\sin\theta_0}\left[\partial_\theta^2 F \partial_\varphi^2 F - \left(\partial_\theta \partial_\varphi F\right)^2\right]^{\frac{1}{2}}\text{, (Supplementary Equation 16)}$$

where, θ, φ are the polar and azimuthal angles, θ_0 is the polar angle of the equilibrium orientation of the magnetization vector, and F is the magnetic free energy of the magnetic material which is composed of various magnetic energies[3]:

$$F = F_0 + F_{dem} + F_a + F_{m.e} + F_{exch} + F_d \quad \text{(Supplementary Equation 17)}$$

where, each energy terms are related to the external magnetic field (F_0), the demagnetization field (F_{dem}), the crystallographic anisotropy (F_a), the magneto-elastic energy ($F_{m.e.}$), the exchange energy (F_{exch}), and the energy of the inter-domain boundary layers (F_d).

For a uniformly magnetized ellipsoidal particle whose magneto-crystalline anisotropy is negligible, it is enough to consider the energy related to the external magnetic field and demagnetization field. By taking the principle axes of ellipsoid to coincide with the coordinate axes, the magnetic free energy has the form:

$$F = -\mathbf{M}\cdot\mathbf{H}_0 + \frac{1}{2}\left(N_x M_{s,x}^2 + N_y M_{s,y}^2 + N_z M_{s,z}^2\right)\text{, (Supplementary Equation 18)}$$

where, N_x, N_y , and N_z are the demagnetizing factors. For a weak or no external magnetic field, the demagnetization energy mainly contributes to the magnetic free energy of the material, and one can find the resonance frequency from supplementary equations (16) and (18) as:

$$\omega_r \approx \gamma\mu_0 M_s \sqrt{\left(N_y - N_x\right)\left(N_z - N_x\right)}\text{, (Supplementary Equation 19)}$$

Then, the resonance frequency thus depends on the demagnetization factor and saturation magnetization of the magnetic material.

For a magnetic thin film having in-plane magnetic domain structures whose width are considerably larger than the thickness of the film, one can assume that demagnetization factor along the surface normal is close to unity while in-plane parts are small and comparable to each other. By taking the normal direction as the z-axis, one can simplify the supplementary equation (19):

$$\omega_r^2 \approx \gamma^2 \mu_0^2 \left(M_s^2 N_x - M_s^2 N_y\right) = \pm 2\gamma^2 \mu_0^2 \left(F_x - F_y\right)\text{, (Supplementary Equation 20)}$$

where the sign is introduced so that the ω_r^2 is positive, and second order terms of N_i are ignored. For a small change of the magnetic domain structure described by a factor $\varepsilon(x,y)$, one can describe the variation of the resonance frequency as:

$$\frac{\Delta\omega_r}{\omega_r} \approx \frac{\gamma^2 \mu_0^2}{F_{xy}} \frac{\partial F_{xy}}{\partial \varepsilon} \varepsilon = \gamma^2 \mu_0^2 \frac{\Delta F_{xy}}{F_{xy}}, \quad F_{xy} = F_x - F_y\text{, (Supplementary Equation 21)}$$

Then, from supplementary equations (15) and (21), one can find a relation between the change of microwave response and magnetic domain structure as:

$$\Delta\chi' = \frac{\chi_0 \gamma^2 \mu_0^2}{2\alpha^2 k^2} \frac{\Delta F_{xy}}{F_{xy}} s, \Delta\chi'' = \frac{\chi_0 \gamma^2 \mu_0^2}{2\alpha^2 k^2} \frac{\Delta F_{xy}}{F_{xy}} s\left(1 - s\right)\text{, (Supplementary Equation 22)}$$

The supplementary equation (22) implies that when a magnetic domain structure changes by an external work, it modifies a magnetic free energy of the domain structure which mainly comes from a change of demagnetization energy of the domain structure. As a result, the response of the microwave changes due to a change of the resonance frequency of the magnetic domain structure.

For example, one can assume a simple case that a micro-strip waveguide is coupled with MDs forming the Landau flux-closure structure as shown in supplementary Fig. 1a. In this case, the response change of the waveguide mainly comes from the precession of two large domains (MD1 and MD2). Here, we assume that the domain wall can shift freely, and the changed MDs by the domain wall shift are stable. When the domain wall is shifted along the x-axis, the resonance frequency of MDs is changed by a variation of demagnetization factor. From supplementary equation (19), one can express the resonance frequency change of each MD induced by the domain wall shift[4]:

$$\omega_{\mathrm{r,MD1}} = \gamma\mu_0 M\sqrt{N_{\mathrm{x}} - N_{\mathrm{y}}} = \omega_{\mathrm{r,0}}\sqrt{1/(1+\varepsilon)}\,,\quad \omega_{\mathrm{r,MD2}} = \omega_{\mathrm{r,0}}\sqrt{1/(1-\varepsilon)}\,,$$

(Supplementary Equation 23)

$$\omega_{\mathrm{r,0}} = \gamma\mu_0 M\sqrt{\pi t / w}\,, \varepsilon = \frac{2\Delta x}{w},$$ (Supplementary Equation 24)

where $\omega_{\mathrm{r,MD1}}$, $\omega_{\mathrm{r,MD2}}$ are the resonance frequency of MD1 and MD2, ε is the domain wall shift, t,w are the thickness and width of the MDs. Supplementary Fig. 1b shows calculated resonance frequency of the MDs (red and black for MD1 and MD2), where one can see that the resonance frequency decreases (increases) along with an increase of domain wall shift (ε). Because the domain wall shift results in a growth of the MD1, while it results in a reduction of MD2, the contribution of them to the response change will be varied. By assuming that the contribution is proportional to the domain width, one can express an effective resonance frequency ($\omega_{\mathrm{r,eff}}$) for a small ε as below:

$$\omega_{\mathrm{r,eff}} = \omega_{\mathrm{r,0}}\left[(1+\varepsilon)\sqrt{1/(1+\varepsilon)} + (1-\varepsilon)\sqrt{1/(1-\varepsilon)}\right] \approx \omega_{\mathrm{r,0}}\left(1-\frac{1}{8}\varepsilon^2\right),$$

(Supplementary Equation 25)

where the approximation was derived with quadratic term second order of ε. As shown in Supplementary Fig. 1b, the $\omega_{\mathrm{r,eff}}$ (green) decrease along with an increase of domain wall shift. Finally, from supplementary equations (14) and (25), we can express the change of imaginary part of magnetic susceptibility by the domain wall shift, which is responsible for the change of microwave absorption of the device, as below:

$$\chi'' = \frac{\chi_0''}{2}\left[1-(1-s_r(\varepsilon)s_0)^2\right],\ s_r(\varepsilon)=\frac{\omega_{r,eff}(0)}{\omega_{r,eff}(\varepsilon)},\ s_0=\frac{\omega}{\omega_{r,eff}(0)},\ \text{(Supplementary Equation 26)}$$

where the s_r and s_0 were introduced to express in terms of normalized frequency, and the αk term in supplementary equation (14) is assumed to be unity to simplify the expression. Supplementary Fig. 1c shows calculated χ'' as a function of ε for s_r=0.9 (blue), 0.985 (red), and 1.0 (black), where the values of s_r are relative microwave frequencies to the maximum $\omega_{r,eff}$. Similar experimental result is presented in Fig. 4j of the manuscript, where one can see the distinct behaviors of χ'' depending on the applied microwave frequency.

Supplementary Note 2: Reversible microwave response modulation by p_m-sweep

Supplementary Fig. 2a-d shows change of real and imaginary parts of $\Delta S_{21,p}$ as a function of p_m for f_m=1.0 GHz, where the p_m was swept from 0 to 30 dBm (forward) and from 30 dBm to 0 dBm (backward), continuously. A change of $\Delta S_{21,p}$ started to occur when the p_m was around 25 dBm during the forward initial p_m-sweep, and the change remained during the subsequent p_m-sweeps. Supplementary Fig. 2e shows line-profiles of real and imaginary parts of the $\Delta S_{21,p}$, where one can see that they showed resonant line-shapes indicating the enhancement of the N-FMR effect. In addition, supplementary Fig. 2f-g shows a change of real part of the $\Delta S_{21,p}$ for a fixed f_p at 1.0 GHz as a function of p_m and sweep index. The line-profile showed that a fluctuation occurred at a high p_m, and the fluctuation decreased as the sweep index increased. Although the $\Delta S_{21,p}$ showed a fluctuation during the p_m-sweep, the fluctuation was negligible compared to the change of $\Delta S_{21,p}$ occurring during the initial p_m-sweep.

Supplementary Fig. 3a-b shows MD images measured at the initial p_m-sweep and at the tenth p_m-sweep. The MD started to change when p_m was around 25 dBm consistent with the $\Delta S_{21,p}$ results. The MDs grew along with an increase of p_m, and the growth MDs showed their initial structure dependency. The grown MDs remained during the subsequent p_m-sweeps, and showed a change of position, width, and local structure (indicated by green circles in 3a-b). Supplementary Fig 3c shows difference of MD images at tenth p_m-sweep, where each images were calculated by subtracting the initial MD image measured at 0 dBm. From the images, one can see that the change of MD structure occurred when p_m exceeds 25 dBm, and the change is maximized at 30 dBm. This result explains well the fluctuation of $\Delta S_{21,p}$ occurring at p_m~25-30 dBm, and indicates that the variation of MD structure is

responsible for the fluctuation. In addition, one can see that there is a part of MD structure (indicated by red circles in 3c-d) showing a reversible change. The reversible change occurred commonly in other p_m-sweeps, where we presented averaged difference of MD images in supplementary Fig. 3d. This result indicates that the MD structure also can be modulated reversible by the p_m-sweep.

While the MD structure showed changes during the p_m-sweep, there was no significant change in the line shapes and amplitude of the $\Delta S_{21,p}$. Supplementary Fig. 3e-f show line-shapes of $\Delta S_{21,p}$ for f_m=1.0 GHz measured at p_m=0 dBm and 30 dBm in tenth p_m-sweep, where one can see that there is no notable difference between the line-shapes even their MD structure are different from each other. In addition, supplementary Fig. 3g shows averaged change of Re-$\Delta S_{21,p}$, where one can see that there is only a slight increase of $\Delta S_{21,p}$ for p_m~25-30 dBm. As the increase of $\Delta S_{21,p}$ means that the microwave absorption is increased, this result can be understood that a higher power of the modulation microwave results in an increase of microwave absorption. However, the increase was comparable to the standard deviation of $\Delta S_{21,p}$ changes, and thus, the MD change result in only a weak variation of the $\Delta S_{21,p}$. We note that although the MD structure changed depending on the p_m, it occurred without a variation of N-FMR frequency. This result can be explained by the MEPP that the change of MD structure occurs maintaining its N-FMR frequency close to the driving frequency, and therefore, the modulated MD structures will give an identical response change even their structure are different from each other.

Supplementary Note 3: *S*-parameter measurement results

Supplementary Fig. 4a shows an illustration on the *S*-parameters of a two port device, where *a* and *b* denote the incident and reflected microwave, and i12 and i21 denote the propagation direction of the modulation and probing microwave. Supplementary Fig. 4b shows an optical image of the device used in the measurement, where the device was connected to a network analyzer (E5071B; Agilent) by 50 Ω BNC cables, and the measurements were conducted by a custom PC program.

Supplementary Fig. 4c and 5a show contour maps of the changes of all *S*-parameters in log magnitude scale as a function of modulation microwave frequency and power, respectively. The measurement results showed that the *S*-parameters were changed strongly occur when the probing and modulation microwave frequencies were around 1.9 GHz.

Supplementary Fig. 6a-b show the change of *S*-parameters around f_p=1.9 GHz as a function of modulation frequency and power, where the left (right) side of the chart shows the measurement result when the modulation microwave was applied from the port 1 to port 2 (from the port 2 to port 1). The measurement results showed that the changes of *S*-parameters can be hysteric depending on the modulation process (see the dotted circle appeared in supplementary Fig. 6a), and they were non-reciprocal with respect to the direction of applied probing and modulation microwaves. Supplementary Fig. 4d-e and 5b-c show contour maps of reciprocity and hysteresis of the *S*-parameters, where one can see that these properties are function of modulation and probing microwave frequency and power.

From these results, we can conclude that there are four parameters modulating the device state: frequency (f_m), power (p_m), and direction (i_m) of modulation microwave, and past excitation state of the device, and the response of the device is determined by two parameters: frequency (f_p), and direction (i_p) of probing microwave. Therefore, kinds of adaptive memory (memristive) operations can be emerged by utilizing such parameters. Supplementary Fig. 6c shows a practical example utilizing the parameters f_m and p_m, where two modulation frequencies of 1.9 GHz (left side of the chart) and 2.1 GHz (left side of the chart) were used to modulate the device state, and the internal state changes were monitored by a probing microwave with a frequency of 1.9 GHz. The measurement result clearly demonstrates that one can realize a continuously tunable adaptive memory operation through suitably chosen modulation parameters.

Finally, we present an implementation of full memristive operation by using two different microwave frequencies. In the present case, the probing process is conducted by measuring the transmitted and reflected power of the modulation microwave rather than applying a weak probing microwave, and two different frequencies having a complementary relation that an internal state changed by one frequency can be reset to the initial state by another frequency are used to realize a reversible memory operation. Supplementary Fig. 6d shows a change of S_{11} and S_{21} as a function of incident microwave power, where two complementary frequencies of 1.9 GHz and 2.1 GHz were used for the memory operations. From the measurement results, one can see that the changes of *S*-parameters caused by an excitation sweep at 1.9 GHz (black) are reset to its initial state after an excitation sweep at 2.1 GHz (red). The present result clearly demonstrates that a reversible memristive operation can be realized from a complementary relation between the excitation frequencies, and therefore,

it can be concluded that the implementation of memristive device operated at microwave frequency can be achieved based on the N-FMR effect of a ferromagnetic insulator.

Supplementary Note 4: Measurement results for initial f_m-sweep in SILPF/YIG/GGG-configuration.

Supplementary Fig. 7b-c shows MD images of yttrium iron garnet (YIG) in initial (before modulation sweep; b) and final (after modulation sweep; c) states in the first f_m-sweep. We note that present MD images do not give information about the magnetization direction of the MDs but give information about the variation of MD structure. Around the high impedance line (HIL), a definite and a localized change of the MD structure appeared after the modulation sweep, while the MD structure far from the HIL were unchanged. The change of MD structure strongly occurred when the f_m was in the range from 1.0 to 2.0 GHz, while the MD structure was nearly unchanged when the f_m was out of that range (supplementary Fig. 7d). Supplementary Fig. 7e-f shows contour maps of transmitted probing microwave power as a function of probing (f_p) and modulation (f_m) frequencies for forward and backward f_m-sweeps. Decreases of transmitted microwave power appeared when the f_p and f_m are both in the range of 1.0 to 2.0 GHz, where the MD structure strongly changed at that modulation frequency range. The coincidence of the frequency range of f_p and f_m showing the microwave transmittance and MD change indicates the modulation of MD and transmittance are caused by N-FMR effect. The overall decrease of transmitted microwave power indicates a population of MDs having a magnetization direction perpendicular to the H-MWNF direction, where the system can get more energy through the spin resonance in that configuration.

Supplementary Note 5: MD modulation process in SILPF/YIG/GGG-configuration

Supplementary Fig. 8 shows a detailed process of MD modulation occurring in f_m from 1.4 GHz to 1.6 GHz with the H-MWNF distribution images, where the results on other excitation frequencies are shown in supplementary Fig. 9. Two kinds of the MD structure changes were observed: a distinct change of MD structure (indicated by green rectangles) around the stubs that remained after the excitations; an appearance of a new MD structure (indicated by red circles) around the high impedance line (HIL) that fluctuated after the excitations (also shown in supplementary Fig. 10b). When the modulation microwave field was applied (ON), the magneto optical (MO) signal around the region showing a fluctuation of the MD structure appeared indistinctly, and it was changed as a function of time as shown

in supplementary Fig. 10a. In particular, by comparing the H-MWNF distributions and MO images, one can see that the indistinct MO signals appear around the intense H-MWNF regions. These results indicate that there is a local instability of MD structure, which is caused by a strong spin precession by a locally intensive H-MWNF, and this instability causes a strong fluctuation of the local MD structure. It is important to note that there is a well defined change of MD structure even in the regions showing a strong fluctuation, as shown in supplementary Fig. 8d that a decrease of MO signal in R_{23} remains after the excitation field is removed (indicated by red circle). This result indicates that a part of MD can be restored even though the excitation causes a strong fluctuation of MD structure, and thus, the reversible modulation of MD structure can be realized from this behavior.

Supplementary Note 6: Jones calculus for thermal stress distribution imaging

From the supplementary Fig. 15c, the Jones matrix for each optical component can be expressed as[5]:

$$\mathbf{J}_{\mathrm{P}} = \begin{bmatrix} 1 & 0 \\ 0 & 0 \end{bmatrix}, \mathbf{J}_{\mathrm{LCM}} = \frac{1}{2}\begin{bmatrix} e^{i\frac{\delta}{2}} + e^{-i\frac{\delta}{2}} & e^{i\frac{\delta}{2}} - e^{-i\frac{\delta}{2}} \\ e^{i\frac{\delta}{2}} - e^{-i\frac{\delta}{2}} & e^{i\frac{\delta}{2}} + e^{-i\frac{\delta}{2}} \end{bmatrix}, \mathbf{J}_{\mathrm{A}} = \begin{bmatrix} \cos^2\phi & \cos\phi\sin\phi \\ \cos\phi\sin\phi & \sin^2\phi \end{bmatrix},$$

(Supplementary Equation 27)

where, δ is the linear birefringence of the LCM, and ϕ is the angle of the analyzer from the x-axis. By assuming no linear and circular dichroism and circular birefringence, the Jones matrix for the sample can be expressed as[6]:

$$\mathbf{J}_{\mathrm{S}} = \begin{bmatrix} e^{i\beta}\cos^2\theta + e^{-i\beta}\sin^2\theta & (e^{i\beta} - e^{-i\beta})\cos\theta\sin\theta \\ (e^{i\beta} - e^{-i\beta})\cos\theta\sin\theta & e^{i\beta}\sin^2\theta + e^{-i\beta}\cos^2\theta \end{bmatrix},$$ (Supplementary Equation 28)

where, β is a linear birefringence induced by thermal stress in the sample, and θ is a angle between the analyzer and the principal stress axis. From the supplementary equations (27) and (28), and from the arrangement of optical components shown in supplementary Fig. 15c, the intensity of the light reaching the CCD can be calculated as:

$$I = \frac{E_{\mathrm{i}}^2}{4}\left(|A|^2\cos^2\phi + |B|^2\sin^2\phi + \left(A^*B + AB^*\right)\cos\phi\sin\phi\right),$$ (Supplementary Equation 29)

where the E_i is the amplitude of electric field of the incident light, and A and B are:

$$A = j_1\delta_+ + j_2\delta_-, \quad B = j_2\delta_+ + j_1^*\delta_-, \text{ (Supplementary Equation 30)}$$

where

$$j_1 = e^{i\beta}\cos^2\theta + e^{-i\beta}\sin^2\theta, \quad j_2 = \left(e^{i\beta} - e^{-i\beta}\right)\cos\theta\sin\theta, \text{ (Supplementary Equation 31)}$$

$$\delta_+ = e^{i\frac{\delta}{2}} + e^{-i\frac{\delta}{2}}, \quad \delta_- = e^{i\frac{\delta}{2}} - e^{-i\frac{\delta}{2}}, \text{ (Supplementary Equation 32)}$$

By choosing the circular polarized light as a probing beam, and from supplementary equations (29)-(32), the output intensity can be calculated as:

$$I_{\phi=\frac{\pi}{2},\delta=-\frac{\pi}{2}} = \frac{E_i^2}{2}\left(1 - \sin 2\beta \sin 2\theta\right), \quad I_{\phi=\frac{\pi}{2},\delta=\frac{\pi}{2}} = \frac{E_i^2}{2}\left(1 + \sin 2\beta \sin 2\theta\right),$$

(Supplementary Equation 33)

$$I_{\phi=\frac{\pi}{4},\delta=-\frac{\pi}{2}} = \frac{E_i^2}{2}\left(1 - \sin 2\beta \cos 2\theta\right), \quad I_{\phi=\frac{\pi}{4},\delta=\frac{\pi}{2}} = \frac{E_i^2}{2}\left(1 + \sin 2\beta \cos 2\theta\right),$$

(Supplementary Equation 34)

and from supplementary equations (33) and (34), and for small β,

$$\beta_1 = \frac{1}{2}\frac{I_{\phi=\frac{\pi}{4},\delta=\frac{\pi}{2}} - I_{\phi=\frac{\pi}{4},\delta=-\frac{\pi}{2}}}{I_{\phi=\frac{\pi}{4},\delta=-\frac{\pi}{2}} + I_{\phi=\frac{\pi}{4},\delta=\frac{\pi}{2}}} \cong \beta\cos 2\theta, \quad \beta_2 = \frac{1}{2}\frac{I_{\phi=\frac{\pi}{2},\delta=\frac{\pi}{2}} - I_{\phi=\frac{\pi}{2},\delta=-\frac{\pi}{2}}}{I_{\phi=\frac{\pi}{2},\delta=-\frac{\pi}{2}} + I_{\phi=\frac{\pi}{2},\delta=\frac{\pi}{2}}} \cong \beta\sin 2\theta,$$

(Supplementary Equation 35)

The β is related to a difference of stress between the two principle axes, and assuming the thermal stress can be described as the plane stress, the stress tensor can be expressed as:

$$\sigma = \begin{bmatrix} \sigma_{\mathrm{x}} & \sigma_{\mathrm{xy}} \\ \sigma_{\mathrm{yx}} & \sigma_{\mathrm{y}} \end{bmatrix} = \begin{bmatrix} \sigma_1\cos^2\theta + \sigma_2\sin^2\theta & (\sigma_1 - \sigma_2)\cos\theta\sin\theta \\ (\sigma_1 - \sigma_2)\cos\theta\sin\theta & \sigma_1\sin^2\theta + \sigma_2\cos^2\theta \end{bmatrix},$$

(Supplementary Equation 36)

where, σ_1 and σ_2 are the two principal axis. From supplementary equation (35) and (36), and from the stress-optic law, the equation (35) can be related to the stress as:

$$\beta_1 = \frac{2\pi dS}{\lambda}\left(\sigma_{\mathrm{x}} - \sigma_{\mathrm{y}}\right),\ \ \beta_2 = \frac{2\pi dS}{\lambda} 2\sigma_{\mathrm{xy}},\ \text{(Supplementary Equation 37)}$$

where S is the stress optical constant, λ is the wavelength of incident light and d is the thickness of the medium.

Supplementary Note 7: Constructing the heat source distribution

For plane strain condition in a rectangular Cartesian coordinate system, the thermal stress can be expressed by introducing the stress function[7]:

$$\sigma_{\mathrm{x}} = \frac{\partial^2\Phi}{\partial y^2} + CT,\ \ \sigma_{\mathrm{y}} = \frac{\partial^2\Phi}{\partial x^2} + CT,\ \ \sigma_{\mathrm{xy}} = -\frac{\partial^2\Phi}{\partial x \partial y},\ \ C = \frac{\alpha E}{1-2v},$$

(Supplementary Equation 38)

where, σ_{x}, σ_{y}, and σ_{xy} are the stress components of the stress tensor, T is the temperature distribution, α, v, and E are the thermal expansion coefficient, the Poisson's ratio and the elastic modulus of a material, and Φ is the stress function satisfying[8]:

$$\nabla^4\Phi = -\frac{\alpha E}{1-v}\nabla^2 T,\ \ \text{(Supplementary Equation 39)}$$

The gradient field of $\nabla^2\Phi$ can be calculated from supplementary equation (38) as:

$$-\frac{\partial(\sigma_{\mathrm{x}} - \sigma_{\mathrm{y}})}{\partial x} - 2\frac{\partial\sigma_{\mathrm{xy}}}{\partial y} = \frac{\partial(\nabla^2\Phi)}{\partial x},\ \ \frac{\partial(\sigma_{\mathrm{x}} - \sigma_{\mathrm{y}})}{\partial y} - 2\frac{\partial\sigma_{\mathrm{xy}}}{\partial x} = \frac{\partial(\nabla^2\Phi)}{\partial y},$$

(Supplementary Equation 40)

and it is related to the LB measurement results from supplementary equations (37) and (40) as:

$$\frac{\partial(\nabla^2\Phi)}{\partial x} = -\frac{\lambda}{2\pi dS}\left(\frac{\partial\beta_1}{\partial x} + \frac{\partial\beta_2}{\partial y}\right),\ \ \frac{\partial(\nabla^2\Phi)}{\partial y} = \frac{\lambda}{2\pi dS}\left(\frac{\partial\beta_1}{\partial y} - \frac{\partial\beta_2}{\partial x}\right),$$

(Supplementary Equation 41)

From the stationary heat equation with a heat source, the heat source distribution can be calculated as:

$$q = -\frac{(1-\nu)k}{\alpha E}\left(\frac{\partial^2\left(\nabla^2\Phi\right)}{\partial x^2}+\frac{\partial^2\left(\nabla^2\Phi\right)}{\partial y^2}\right),\quad q = k\nabla^2 T \text{ , (Supplementary Equation 42)}$$

where, q is the heat source density, and k is the effective thermal conductivity of the platinum coated glass substrate. Finally, from supplementary equations (41) and (42), the heat source distribution can be expressed as:

$$q = \frac{\lambda}{2\pi dS}\frac{(1-\nu)}{\alpha Ek}\left(2\frac{\partial^2\beta_2}{\partial x\partial y}+\frac{\partial^2\beta_1}{\partial x^2}-\frac{\partial^2\beta_1}{\partial y^2}\right),\quad \text{(Supplementary Equation 43)}$$

Supplementary Fig. 16a-b shows a calculated heat distribution images by supplementary equation (43) and measured temperature distribution image by IR-camera, where a PCB circuit under DC current of 1A was used as the device under test, and a platinum coated (t=200nm) glass substrate was used as the indicator. As expected, the calculation result well describes the heat source distribution of the PCB circuit. Supplementary Fig. 16c shows magnetic microwave near field (H-MWNF) distribution image measured by the present method. From the measurement result, one can see that intense regions appear around the high impedance line (HIL). This result indicates that the H-MWNF is strong around the HIL, and it is a common feature of the SILPFs.

SUPPLEMENTARY REFERENCES

1. Pain, D. *et al.* An improved permeameter for thin film measurements up to 6 GHz. *J. Appl. Phys.* **85,** 5151 (1999).
2. Pozar, D. M. Microwave Engineering. 4th ed. New York: Wiley (2011).
3. Vonsovskii, S. V. Ferromagnetic resonance. Pergamon press (1966).
4. Krasyuk, A. *et al.* Self-Trapping of Magnetic Oscillation Modes in Landau Flux-Closure Structures. *Phys. Rev. Lett.* **95,** 207201 (2005).

5. Ishibashi, T., *et al.* Magneto-optical imaging using polarization modulation method. *J. Appl. Phys.* **100**, 093903 (2006).
6. Xie, X., Simon, J. D. Picosecond circular dichroism spectroscopy: a Jones matrix analysis. *J. Opt. Soc. Am. B* **7**, 1673 (1990).
7. Ainola, L., Aben, H. On the generalized Wertheim law in integrated photoelasticity. *J. Opt. Soc. Am. A* **25**, 1843 (2008).
8. Barron, R. F., Barron, B. R., *Design for Thermal Stresses* Ch. 6 (Wiley, 2011).
